# Antifungal and Cytotoxic Activity of Diterpenes and Bisnorsesquiterpenoides from the Latex of *Euphorbia resinifera* Berg

**DOI:** 10.3390/molecules27165234

**Published:** 2022-08-16

**Authors:** El-Mahdi Ourhzif, Alessandra Ricelli, Venturina Stagni, Angela Cirigliano, Teresa Rinaldi, Latifa Bouissane, Luciano Saso, Pierre Chalard, Yves Troin, Mostafa Khouili, Mohamed Akssira

**Affiliations:** 1 Institut de Chimie de Clermont-Ferrand (ICCF), Dep. Chimie Organique et Medicinale, Université Clermont Auvergne CNRS SIGMA, F-63000 Clermont-Ferrand, France; 2 Laboratoire de Chimie Physique et Biotechnologie des Biomolécules et des Matériaux, Faculté des Sciences et Techniques, Université Hassan II Casablanca, BP 146, Mohammedia 28800, Morocco; 3 Laboratoire de Chimie Moléculaire, Matériaux et Catalyse (LCMMC), Faculté des Sciences et Techniques, Université Sultan Moulay Slimane, BP 523, Beni-Mellal 23000, Morocco; 4 Institute of Molecular Biology and Pathology-CNR, P.le Aldo Moro, 5, 00185 Rome, Italy; 5 Department of Biology and Biotechnology, “Charles Darwin” Sapienza University, P.le Aldo Moro, 5, 00185 Rome, Italy; 6 Department of Physiology and Pharmacology, “Vittorio Erspamer” Sapienza University, P.le Aldo Moro, 5, 00185 Rome, Italy

**Keywords:** *Euphorbia resinifera*, diterpenes, bisnorsesquiterpenoids, biological effect, *Saccharomyces cerevisiae*, *Aspergillus carbonarius*, breast cancer cell line

## Abstract

*Euphorbia resinifera* latex has been extensively utilized in traditional medicine due to its range of bioactivities. Chromatographic separations on silica gel of ethanol extract of *E. resinifera* latex led to the development of a new procedure for isolating resiniferatoxin (**4**) via dried *E. resinifera* latex and the identification of nine compounds. Among these, catechol (**7**), protocatechuic acid (**8**) and 3,4-dihydroxyphenylacetic acid (**9**), known phenolic compounds, were identified for the first time in *E. resinifera* latex. Herein we investigated the effects of major compounds of the latex of *E. resinifera* on the yeast *Saccharomyces cerevisiae*, on the growth of *Aspergillus carbonarius*, a widespread fungal contaminant, and on the breast cancer cell line MCF7 as well as on MCF10A normal breast cells. 12-deoxyphorbol-13-isobutyrate-20-acetate (**2**) had an inhibiting effect on the growth of *A. carbonarius*, and 7-*p*-metoxyphenylacetate-3,8,12-triacetate ingol (**3**) showed a negative effect on yeast cell growth and also a cytotoxic effect on breast cancer cell line MCF7, but not on MCF10A cells. Deglucosyl euphorbioside A (**5**) and euphorbioside A (**6**) showed a discoloration effect that was possibly related to mitochondrial functionality in yeast, and also cytotoxicity only on the cancer cell line that was tested. Interestingly, treatment of MCF7 cells with 7-*p*-metoxyphenylacetate-3,8,12-triacetate ingol (**3**) and deglucosyl euphorbioside A (**5**) not only led to a specific cytotoxic effect but also to the increase in the level of intracellular ROS.

## 1. Introduction

According to the World Health Organization, cancer was responsible for approximatively 9.6 million deaths worldwide, with 18.1 million new cases recorded in 2018 [1]; among those new cases, lung and breast cancers are prevalent. Breast cancer is the most frequently diagnosed and its incidence far exceeds that of other cancers in both developed and developing countries [2]. One major form of cancer treatment involves chemotherapy by means of synthetic drugs [3]. However, critical side-effects (from vomiting and diarrhea, to more major complications e.g., neurological, cardiac, pulmonary, and renal toxicity) and the development of multi-drug resistance (MDR), are often associated with these drugs and can lead to poor therapeutic efficiency. Likewise, the incidence of fungal infections is a major cause of morbidity and mortality in immunocompromised patients worldwide [2]. The relative lack of available antifungal compound classes and the resistance of certain fungal species to the available drugs are obstacles to treating these infections [4]. Thus, there is a significant demand for new drugs with improved potency and lower toxicity and there is continuous research to find new anticancer and antifungal agents in both academic and industrial settings [3].

Natural molecules with antifungal and anticancer properties have been a key research focus over the past few years. Recently, natural products have proven to be a key source of new drugs with a wide range of biological and pharmacological applications [5,6]. The Euphorbiaceae family is ubiquitous and pharmaceutically relevant. In particular, the genus *Euphorbia* is the most important genus of the Euphorbiaceae family, with more than 2000 species worldwide, all of which are characterized by the presence of milky irritant latex [7].

*Euphorbia resinifera* Berg. is a plant that is endemic to Morocco, where it typically grows on the slopes of the anti-Atlas Mountains [8]. *Euphorbia resinifera*, and specifically its latex, has been studied primarily due to the presence of diverse phytochemicals such as polycyclic and macrocyclic, diterpenes [8,9,10,11,12], triterpenes [13,14,15], sesquiterpenoids [8], and phenolic acids [16]. These chemical constituents are providing leading compounds for drug discovery due to their therapeutic applications deriving from their cytotoxic [15,17,18,19], anti-neurodegenerative [20], antiviral [21,22], antimicrobial [23], and antiparasitic [24] properties and anti-inflammatory activities [25,26]. Euphorbium- i.e., the dried latex of *E. resinifera* contains a daphnane diterpene (+)-Resiniferatoxin (RTX), one of the most ancient drugs that is still being used as a starting point in the development of a novel class of analgesics [27,28,29]. RTX is used in the treatment of pain that is associated with diabetic polyneuropathy and in the desensitization of nociceptive neurons [30,31,32]. This compound also shows a varied biological response and has powerful analgesic effects that are more potent than capsaicin (at least 10^3^–10^5^ times) [33]. Furthermore, Euphorbium is used in Moroccan traditional medicine to suppress chronic pain, to mitigate pain for dental cavities and tooth aches, and treat articular tuberculosis [34]. As part of our continuing valorization of *Euphorbia* species that are native to Morocco, we have investigated the effects of the major compounds of the latex of *E. resinifera* on the growth of the yeast *Saccharomyces cerevisiae* and of *Aspergillus carbonarius*, a widespread fungal contaminant; furthermore, the cytotoxic effect was evaluated on breast cancer cell line MCF7, as well as on normal breast cells MCF10A.

## 2. Results

### 2.1. Chemical Results

**Figure 1:**
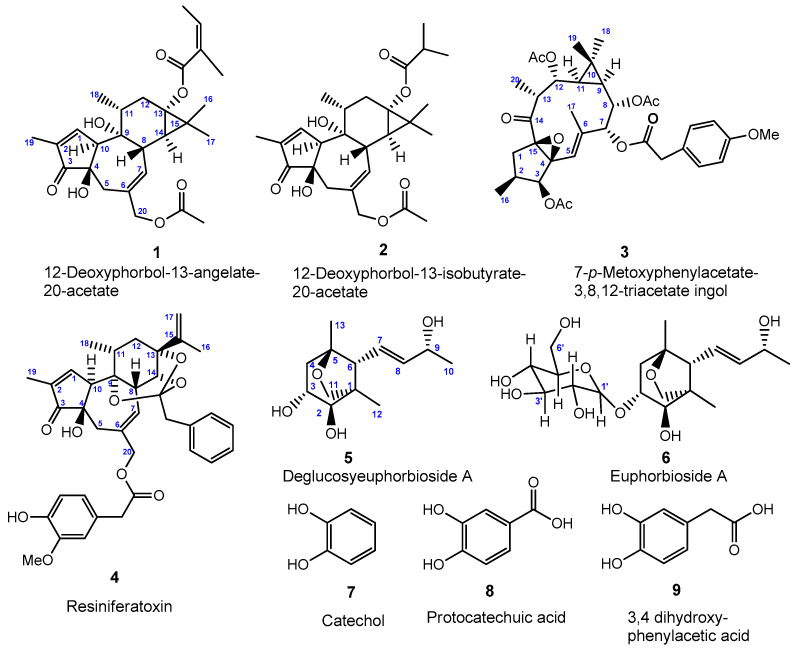
Chemical structure of compounds **1**–**9** that were isolated from the latex of *Euphorbia resinifera*.

Within the context of our search for the chemical constituents of *Euphorbia resinifera* latex and their antifungal and cytotoxic activities, the dried *E. resinifera* latex was extracted with ethanol, employing a Soxhlet apparatus. The combined extracts were concentrated to produce the ethanol extract. The extract was processed with increasing polarity liquid-liquid partition and continuous conventional chromatographic technique combination, such as normal-phase silica gel column chromatography and preparative normal-phase thin-layer chromatography (TLC). A total of nine compounds were characterized and among them certain phenolic compounds, catechol (**7**), protocatechuic acid (**8**), and 3,4 dihydroxy-phenylacetic acid (**9**) (Figure 1) were identified for the first time in *E. resinifera* latex. Other known compounds were also identified based on the spectral and chemical evidence, which were in agreement with those that were reported in the literature [7].

The *n*-hexane extraction resulted in the efficient removal of the major triterpenes from the latex, which potentially contains α-euphol and α-euphorbol derivatives [14,24]. From the dichloromethane fraction we have isolated four major diterpenes in the latex of *E. resinifera*. There were two phorbol esters, 12-deoxyphorbol-13-angelate-20-acetate (**1**) [8,10,17] and 12-deoxyphorbol-13-isobutyrate-20-acetate (**2**) [8,35] that were identified, in addition one component that was structurally related to the ingol skeleton, 7-*p*-metoxyphenylacetate-3,8,12-triacetate ingol (**3**) [11] was also isolated. The remaining compound was identified as resiniferatoxin (**4**). Attempts to isolate resiniferatoxin from *E. resinifera* latex were eventually successful; no resiniferatoxin could be isolated from the diterpenoid fraction that was obtained from Euphorbium. Corresponding data for resiniferatoxin are compared with natural and synthetic resiniferatoxin [10,27,29,36].

The *n*-butanol fraction was fractionated and purified by column chromatography. There were two major norsesquiterpenoid compounds that were isolated, deglucosyl euphorbioside A (**5**) and euphorbioside A (**6**); these compounds have already been reported as having been present in *E. resinifera* [8]. The remaining fraction of ethyl acetate yielded three known phenolic compounds, catechol (**7**) [37], protocatechuic acid (**8**) [38], and 3,4 dihydroxy-phenylacetic acid (**9**) [39]. To the best of our knowledge, this is the first time these compounds have been identified in *E. resinifera* latex.

Inspired by the study of phytochemical and anti-cancer activity of a plant belonging to the genus *Euphorbia* that was conducted by Munro et al. [40], we tested 12-deoxyphorbol-13-angelate-20-acetate (**1**), 12-deoxyphorbol-13-isobutyrate-20-acetate (**2**), 7-*p*-metoxyphenylacetate-3,8,12-triacetate ingol (**3**), deglucosyl euphorbioside A (**5**), and euphorbioside A (**6**) (Figure 1) to evaluate their effects on the yeast *Saccharomyces cerevisiae*, on *Aspergillus carbonarius* growth, on the MCF7 breast cancer cell line, as well as on the MCF10A normal breast cells.

### 2.2. Biological Results

#### 2.2.1. Evaluation of the Effect of Compounds on the Yeast *S. cerevisiae*

**Figure 2:**
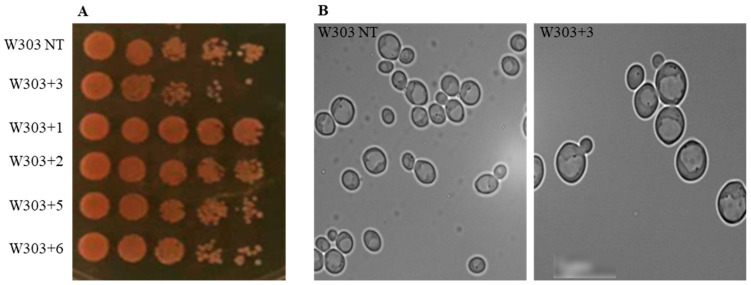
Effects of the tested compounds on *S. cerevisiae* W303 exponential growth phase. (**A**): yeast cells that were treated with compounds at 400 μM; 7-*p*-metoxyphenylacetate-3,8,12-triacetate ingol (**3**) showed a negative effect on yeast cell growth, while 12-deoxyphorbol-13-angelate-20-acetate (**1**) had a positive effect. (**B**): Cells that were treated with compound **3** showed a bigger size with swollen vacuoles. The experiment was conducted in three independent replicates.

The yeast cells were treated with the considered compounds at 400 μM (Figure 2A) [41]; the concentration of 400 μM was selected after carrying out a dose response curve with concentrations from 10 μM to 500 μM (not shown). The 7-*p*-metoxyphenylacetate-3,8,12-triacetate ingol (**3**) was found to exert a negative effect on yeast growth. On the contrary, 12-deoxyphorbol-13-angelate-20-acetate (**1**) showed a positive effect. The observation of the culture showed that the cells that were treated with 7-*p*-metoxyphenylacetate-3,8,12-triacetate ingol (**3**) evidenced a bigger cell size with swollen vacuoles, compared to untreated yeast cells (W303 NT) (Figure 2B).

**Figure 3:**
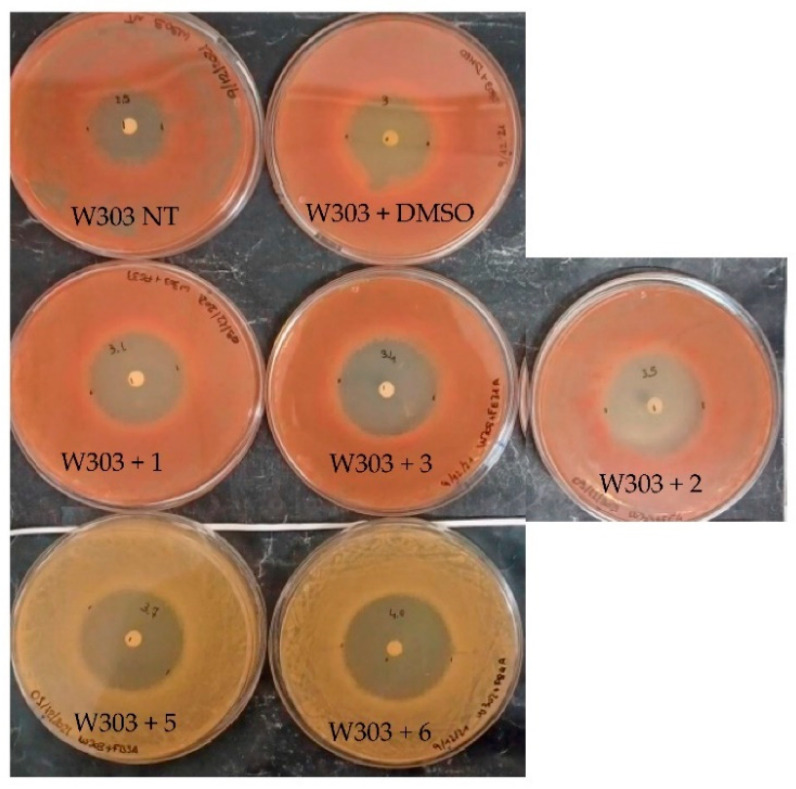
H_2_O_2_ sensitivity of *S. cerevisiae* that was induced by the tested compounds. The sensitivity was visualized with a growth inhibition test. Deglucosyl euphorbioside A (**5**) and euphorbioside A (**6**) induced a slight sensitivity to H_2_O_2_ producing a halo of 3.7 cm and 4 cm, respectively, bigger than the halo that was produced with cells that were treated with DMSO (3 cm).

To verify if the cells that were treated with the compounds overproduced ROS, cells were stained with dihydrorodamine123. In the tested conditions, the compounds did not induce oxidative damage in the yeast cells, because no evident overproduction of ROS was visualized. In fact, most of the treated cells in the logarithmic phase showed a limited fluorescence, reflecting the presence of normal ROS background (data not shown). We then evaluated by halo test the sensitivity of the treated cells to hydrogen peroxide (H_2_O_2_). The yeast cells were treated with the compounds at 400 μM and plated in YPD medium. A paper disk was soaked with 5 μL of H_2_O_2_ 9.2 M. After 24 h, the diameter of the halo was measured (Figure 3). In the cells that were treated with deglucosyl euphorbioside A (**5**) and euphorbioside A (**6**), a higher sensitivity to H_2_O_2_ compared to the cells that were challenged only with dimethyl sulfoxide (DMSO) was observed, with a halo production of 3.7 cm and 4 cm, respectively, instead of 3 cm (Figure 3). It is also interesting to note the loss of the red color of the yeast cells that were treated with these compounds, see “Discussion”.

#### 2.2.2. Evaluation of the Compounds’ Antifungal Effect

**Figure 4:**
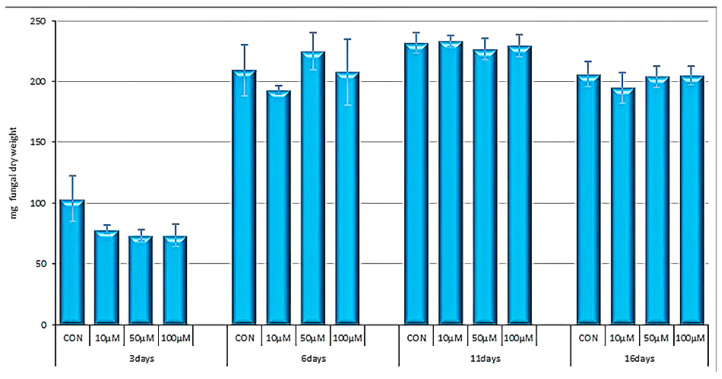
The effect of 12-deoxyphorbol-13-isobutyrate-20-acetate (**2**) on the growth of *Aspergillus carbonarius*. The fungus was cultured in CDY 0.2% at 25 °C. The data represent the average with standard deviations from three independent experiments. The results are expressed as percentages of growth normalized to untreated fungal culture (CON).

The considered compounds were also assayed on the fungus *A. carbonarius* at 10 μM, 50 μM, and 100 μM (Figure 4). The growth of the fungus was investigated after 3, 6, 11, and 16 days of incubation, i.e., between the beginning of the exponential growth phase and the plateau phase. Adding 12-deoxyphorbol-13-isobutyrate-20-acetate (**2**) at all the tested concentrations to the fungal culture had the effect of decreasing its growth by 25%. This effect, evident after 3 days of incubation, was lost after longer incubation periods. The remaining compounds that were tested did not display any significant effect on fungal growth.

#### 2.2.3. Evaluation of the Compounds’ Cytotoxicity

**Figure 5:**
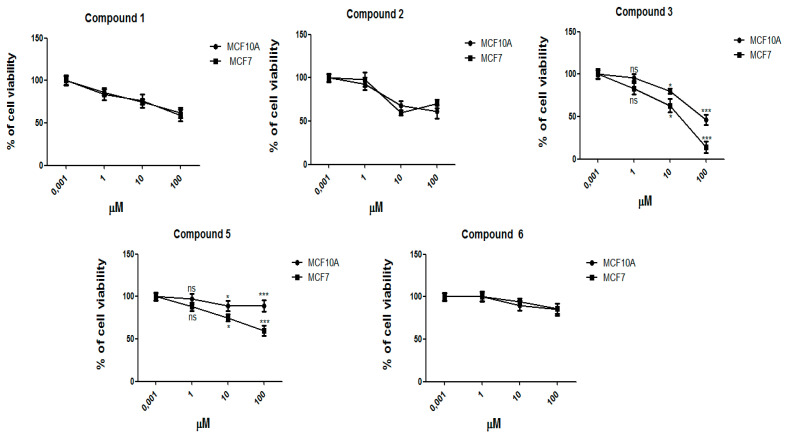
Comparison of the cell viability of MCF10A and MCF7 cell lines that were treated for 72 h with different concentrations of compounds (0.001, 1, 10, and 100 μΜ). Cell viability was measured by MTS assay. The line-graphs represent the average with standard deviations from three independent experiments. Results are expressed as the percentage of cell viability normalized to the untreated cells. Statistical differences between MCF7 and MCF10A cells were assessed by Student’s *t*-test (* *p* < 0.05, *** *p* < 0.001), ns = not significant.

To explore the effect of the main compounds that were obtained from the *E. resinifera* extracts on breast cancer and normal cell growth, the cells were exposed to different concentrations (0.001 µM, 1 µM, 10 µM, and 100 µM) of 12-deoxyphorbol-13-angelate-20-acetate (**1**), 12-deoxyphorbol-13-isobutyrate-20-acetate (**2**), 7-*p*-metoxyphenylacetate-3,8,12-triacetate ingol (**3**), deglucosyl euphorbioside A (**5**), and euphorbioside A (**6**) for 72 h, and the cell viability was assessed as described in Figure 5. Our results have shown a significant decrease in the percentage of viable breast cancer cells MCF7 that were treated with increasing concentrations of the considered molecules (Figure 5). This result was particularly evident for 7-*p*-metoxyphenylacetate-3,8,12-triacetate ingol (**3**). To investigate whether the cytotoxicity of the compounds is specific for breast cancer cells, we treated with the same doses also normal breast cells MCF10A. The subsequent cytotoxic effect was significantly lower for 7-*p*-metoxyphenylacetate-3,8,12-triacetate ingol (**3**) and deglucosyl euphorbioside A (**5**) at 10 µM and 100 µM, compared to the breast cancer cells MCF7, suggesting that these compounds are more selective to cancer cells than normal ones (Figure 5).

#### 2.2.4. Evaluation of Mitochondrial ROS Formation in Human Cell Lines

**Figure 6:**
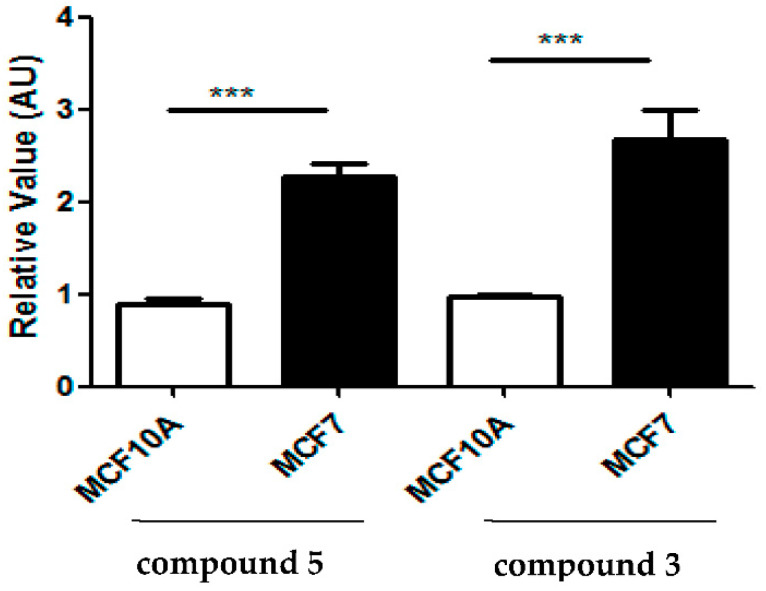
Analysis of mitochondrial ROS using the dye MitoSox Red in cells that were incubated 1 h with 7-*p*-metoxyphenylacetate-3,8,12-triacetate ingol (**3**) and deglucosyl euphorbioside A (**5**) at 10 μM. The data are represented with fold of induction compared to the untreated samples (relative value). All the values are expressed as the average with standard deviations from three independent experiments. Statistical differences between MCF7 and MCF10A cells that were treated at 10 µM were assessed by Student’s *t*-test (*** *p* < 0.001).

To investigate the possible generation of ROS in response to the tested compounds, MCF7 and MCF10A cells were treated with 7-*p*-metoxyphenylacetate-3,8,12-triacetate ingol (**3**) and deglucosyl euphorbioside A (**5**) to obtain 10 μΜ concentration and the level of ROS production was assessed accordingly (Figure 6). A significant increase in the level of intracellular ROS was observed in MCF7 cells that were treated with 7-*p*-metoxyphenylacetate-3,8,12-triacetate ingol (**3**) and deglucosyl euphorbioside A (**5**). However, the level of ROS production remained approximately unchanged in MCF10A cells (fold change compared to the untreated cells) (Figure 6).

Overall, these data suggest a selective cytotoxic effect of 7-*p*-metoxyphenylacetate-3,8,12-triacetate ingol (**3**) and deglucosyl euphorbioside A (**5**) on breast cancer cells, probably due to increasing ROS production.

## 3. Discussion

The main compounds that were purified from the Euphorbium (Figure 1) were tested in different contexts: yeast cells, a representative of the mold species contaminating various foodstuffs, and cancerous and normal cells. Each one of the tested organisms and cell lines has its own specificity. In particular, *S. cerevisiae* constitutes a valid model for evaluating the effects of molecules that are endowed with biological activity and it is essential to investigate the effects on the mitochondrial function because the mitochondrial respiration is dispensable in *S. cerevisiae* [42,43,44].

The considered compounds were tested at 400 μM because this was the minimal concentration showing a toxic effect. The use of compounds at the concentration of 400 uM is not unusual for natural compounds, also given the presence of the thick, rigid cell wall of the yeast *S. cerevisiae* [41,43].

The effect of the tested compounds on the yeast *S. cerevisiae* revealed that the 7-*p*-metoxyphenylacetate-3,8,12-triacetate ingol (**3**) exerted a negative effect on yeast growth inducing a bigger cell size with swollen vacuoles (Figure 2), while deglucosyl euphorbioside A (**5**) and euphorbioside A (**6**) induced sensitivity to H_2_O_2_. It is interesting to note that deglucosyl euphorbioside A (**5**) was also active in MCF7 cells, inducing ROS production. Moreover, deglucosyl euphorbioside A (**5**) and euphorbioside A (**6**) negatively influenced the accumulation of an adenine biosynthetic pathway intermediate because the treated cells lost the red color (Figure 3) characteristic of a defective adenine biosynthesis in *S. cerevisiae* cells [45]. The loss of the red color could also indicate a mitochondrial dysfunction because the W303 wild-type yeast colonies are red because the ade2^-^ mutation results in the accumulation of the substrate of the Ade2, an enzyme of the adenine biosynthetic pathway. The formation of this red intermediate is produced in the mitochondria. If the mitochondria are not functional, the intermediate is not produced, and the colonies result in white colonies. Thus, yeast cells that were treated with 7-*p*-metoxyphenylacetate-3,8,12-triacetate ingol (**3**), deglucosyl euphorbioside A (**5**), and euphorbioside A (**6**) deserve further analysis.

The test of the molecules that were considered on the fungus *A. carbonarius* allowed to evaluate the possible effect on the growth of this widespread contaminant. In fact, the inhibitory effect of plant extracts from the genus *Euphorbia* against *Aspergillus niger*, a fungal contaminant belonging to the “black Aspergilli” group as well as *A. carbonarius*, has been demonstrated [46,47].

The only compound that had an inhibitory effect on the growth of *A. carbonarius* was 12-deoxyphorbol-13-isobutyrate-20-acetate (**2**) (Figure 4). This molecule only differs from the ineffective compound 12-deoxyphorbol-13-angelate-20-acetate (**1**) in the ester group chain, which makes molecule 1 larger and planar. We can hypothesize that the general structure of the molecule is a good starting point for the search for compounds that are capable of inhibiting fungal growth. It is possible that the lack of effect of molecule 1 on fungal growth may be due to its steric hindrance, which does not allow the interaction with the membrane receptors or the passage of the membrane itself.

It has been reported that some species of *Euphorbia* may have cytotoxic effects against different cell lines [48]. In particular, the in vitro anti-cancer and genotoxic activity of methanolic extract of *Euphorbia triaculeata* were previously evaluated using the MTT assay in human breast cancer cell line MCF7 and normal breast epithelial cell line MCF10A [49]. Thus, we decided to use these cell lines to test the cytotoxic effects of our compounds by comparing the behavior of tumor and normal cell lines (Figure 5). A panel of concentrations was tested to obtain a general evaluation of the anti-cancer effects of our compounds. Future experiments, in which we will compare our compounds with well-known anti-cancer compounds, could clarify their anti-cancer activity.

This kind of testing leads to the selection of compounds to be sent for further experimentation and finally to a possible use as therapy.

An increase in intracellular ROS level in cancer cells sustains stressful conditions [50]. The role of ROS in breast cancer is very controversial. It is well known that breast cancer cell lines MCF7 cells and non-transformed breast cell lines MCF10A express low levels for ROS [48]. Interestingly, the induction of ROS in breast cancer cell lines MCF7 by several drug treatments is correlated with a loss of cell viability (Figure 5), suggesting a role of ROS induction as a mechanism of apoptosis induction in cancer cells [51]. Consistently, we found that 7-*p*-metoxyphenylacetate-3,8,12-triacetate ingol (**3**) and deglucosyl euphorbioside A (**5**) were able to increase mitochondrial ROS levels in MCF7 cells, but not in the non-transformed MCF10A cells (Figure 6), suggesting that the loss of viability that is induced by these two drugs is related to the difference in ROS induction, but only at the highest assayed doses. These results point out that these compounds are cytotoxic on cancer cells, albeit only at high doses, suggesting that they are not useful as such for anti-cancer therapies. Further experiments will clarify this point.

It is interesting to note how 12-deoxyphorbol-13-isobutyrate-20-acetate (**2**) has shown activity on both *A. carbonarius* growth and on higher cells, despite the diversity of these biological systems. Furthermore, the loss of the red color of the yeast cells that were treated with deglucosyl euphorbioside A (**5**) and euphorbioside A (**6**) is a clue that encourages studies on the effect of these compounds on mitochondrial functionality.

Overall, our data characterized for the first time the effects of these natural compounds in different systems.

## 4. Materials and Methods

### 4.1. General Experimental Procedures

Analytical and preparative normal-phase thin-layer chromatography (TLC) were performed on 100 mm × 200 mm glass that was pre-coated with 0.25 mm layer of silica plates Kiesilgel 60F254 (Merck, Darmstadt, Germany) for analytical TLC, and with 0.5 mm layer of silica gel for preparative TLC. Spots were visualized using short (254 nm) UV light before using an ethanolic solution of phosphomolybdic acid (heating). Purifications by column chromatography were performed using silica gel Kiesgel 60, 40−63 μm. The melting points (mp) were measured by a Kofler hot bench or Reichert plate-heating microscope and are reported uncorrected. The IR spectra were obtained using IRAffinity-1S FTIR Infrared spectrophotometer (Shimadzu, Kyoto, JP). The measurements were made by loading the sample directly onto a diamond cell. The measurements are reported on the wavenumber scale (cm^−1^). ^1^H and ^13^C NMR spectra were recorded on a BrükerAvance spectrometer (Billerica, MA, USA) at 400.13 and 100.61 MHz, respectively, using CDCl_3_ and CD_3_OD-d4 as solvent. Chemical shifts (^1^H and ^13^C) were reported in ppm relative to the solvent residual signals (e.g., chloroform: δ^1^H = 7.26 ppm and δ^13^C = 77.16 ppm; methanol: δ^1^H = 4.87 ppm and δ^13^C = 49.00 ppm). The spectra were processed with Mnova program from MestRelab Research. LC−MS was recorded a Q Exactive Quadrupole-Orbitrap mass spectrometer that was coupled to an HPLC Ultimate 3000 that was equipped with a DAD UV/vis 3000 RS detector (Thermo Fisher Scientific, Waltham, MA, USA. The column was a Kinetex EVO C18; 1.7 μm; 100 mm × 2.1 mm. A flow rate of 0.45 mL min^−1^ was applied with the following linear gradient: solvent B from 5% to 95% over 7.5 min (solvent A = H_2_O + 0.1% formic acid, solvent B = acetonitrile + 0.1% formic acid).

### 4.2. Plant Material

Latex from *E. resinifera* Berg. was collected in October 2016, from plants in the area of Azilal, Morocco, by making repeated cuts along the stems of the plants with a knife and collecting the white milky exudates. A voucher specimen (20161023) deposited at the herbarium of Laboratory of Molecular Chemistry, Materials, and Catalysis, Sultan Moulay Slimane University, Faculty of Science and Technology, BP 523, 23,000 Beni-Mellal.

### 4.3. Extraction and Isolation

The latex (1.5 L) from *E. resinifera* Berg. was air-dried at room temperature in a dark room, and the resulting coagulum (600 g) was extracted with 96% EtOH (3 L), employing a Soxhlet apparatus. After 24 h, ethanolic extract was concentrated to obtain a crude gummy material (88.8 g). This crude extract was then dissolved in water and extracted three times with increasing polarity solvent to give five fractions: *n*-hexane (23.5%), CH_2_Cl_2_ (24%), EtOAc (1.7%) *n*-butanol (2.3%), and water (48.5%).

Part of the CH_2_Cl_2_ fraction (20 g out of 20.9 g) was subjected to normal-phase silica gel column chromatography that was eluted with a gradient of cyclohexane-EtOAc (from 100:0 to 0:100, *v*:*v*) to yield seven major fractions (1−7). Fraction 5 (3.1 g) was then partitioned through a silica gel column chromatography with cyclohexane-EtOAc as eluent (100:0 then 50:50, *v*:*v*) to afford six subfractions (5A to 5F). Subfraction 5C (270 mg) was purified by column chromatography over normal-phase silica gel that was eluted with cyclohexane-EtOAc (100:0 to 80:20, *v*:*v*) to give 12-deoxyphorbol-13-angelate-20-acetate (**1**) (32 mg) and 7-*p*-metoxyphenylacetate-3,8,12-triacetate ingol (**3**) (25 mg). Subfraction 5D (200 mg) was purified with a silica gel chromatography column eluting with cyclohexane-EtOAc (60:40, *v*:*v*) to obtain 12-deoxyphorbol-13-isobutyrate-20-acetate (**2**) (90 mg). Subfraction 5E (130 mg) was fractionated using normal-phase column chromatography and cyclo-hexane-EtOAc (85:15 to 68:32, *v*:*v*) as a mobile phase, which gave two fractions (5E1 and 5E2). Subfraction 5E2 (85 mg) was purified by preparative TLC with cyclohexane-EtOAc (68:32, *v*:*v*) to afford resiniferatoxin (**4**) (10 mg).

Part of the *n*-butanol fraction (1.8 g out of 2 g) was subjected to silica gel column chromatography using solvent mixture of EtOAc-MeOH (0:100 to 80:20, *v*:*v*) to obtain five fractions (1–5). Fraction 3 (230 mg) was chromatographed on a silica gel column eluting with EtOAc-MeOH (90:10, *v*:*v*) to yield deglucosyl euphorbioside A (**5**) (40 mg). Fraction 4 (350 mg) was fractionated over a silica gel column and eluted with a gradient of EtOAc-MeOH (from 0:100 to 70:30, *v*:*v*) to give two fractions (4A et 5B). Subfraction 4A (150 mg) was further purified by column chromatography over normal-phase silica gel that was eluted with EtOAc-MeOH (80:20, *v*:*v*) to furnish euphorbioside A (**6**) (70 mg).

The EtOAc fraction (1.5 g) was partitioned on a silica gel column and eluted with cyclohexane-EtOAc (from 90:10 to 0:100) to yield five fractions (1–5). Fraction 2 (14.7 mg) was further subjected to a normal-phase column chromatography with cyclohexane-EtOAc (75:25, *v*:*v*) as an eluent to afford catechol (**7**) (10 mg). Fraction 3 (100 mg) was further purified using silica gel column chromatography with cyclohexane-EtOAc as an eluent (90:10, *v*:*v*) to produce protocatechuic acid (**8**) (50 mg). Fraction 4 (200 mg) was further chromatographed over a silica gel column chromatography and eluted with cyclohexane-EtOAc (90:10, *v*:*v*) to yield 3,4-dihydroxyphenylacetic acid (**9**) (100 mg).

### 4.4. Physical and Spectroscopic Data of Compounds ***1***–***9***

#### 4.4.1. 12-Deoxyphorbol-13-angelate-20-acetate (**1**)

Light-yellow oil; Rf = 0.26 (20% EtOAc/hexane); ^1^H NMR (400 MHz, CDCl_3_, δ ppm, *J* Hz): δ = 7.63 (br s, 1H, H-1), 6.17 (q, 1H, *J* = 7.3 Hz, angelate), 5.88 (s, 1H, OH-9), 5.74 (d, 1H, *J* = 5.4 Hz, H-7), 4.46 (q, 2H, *J* = 12.2 Hz, H2-20), 3.31 (br s, 1H, H-10), 3.00 (t, 1H, *J* = 5.4 Hz, H-8), 2.52 (d, 1H, J = 19.0 Hz, H-5α), 2.39 (d, 1H, J = 19.0 Hz, H-5β), 2.22 (br s, 1H, OH-4), 2.08 (dd, 1H, *J* = 14.3, 7.1 Hz, H-12α), 2.05 (s, 3H, CH_3_CO), 2.02 (m, 1H, H-11), 1.79 (br s, 3H, H3-19), 1.86 (br s, 3H, angelate), 1.87 (d, 3H, *J* = 7.3 Hz, angelate), 1.63 (dd, 1H, *J* = 14.4, 11.1 Hz, H-12β), 1.25 (s, 3H, H3-16), 1.09 (s, 3H, H3-17), 0.9 (d, 3H, *J* = 6.4 Hz, H3-18), 0.87 (d, 1H, *J* = 5.4 Hz, H-14). ^13^C NMR (101 MHz, CDCl_3_, δ ppm): δ = 209.2, 170.9, 169.6, 161.6, 141.2, 134.9, 134.2, 133.0, 127.5, 76.2, 73.8, 69.9, 63.3, 55.9, 39.7, 39.2, 36.6, 32.9, 32.1, 23.8, 23.1, 21.1, 20.6, 18.7, 16.2, 15.5, 10.3. HRMS-ESI (*m*/*z*): calcd for C_27_H_36_O_7_ [M-H]^−^ 471.2388; found 471.2389, t_R_ = 6.37 min.

#### 4.4.2. 12-Deoxyphorbol-13-isobutyrate-20-acetate (**2**)

Colorless oil; R*_f_* = 0.56 (32% EtOAc/hexane); ^1^H NMR (400 MHz, CDCl_3_, δ ppm, *J* Hz): δ = 7.60 (br s, 1H, H-1), 5.72 (d, 1H, *J* = 5.4 Hz, H-7), 5.61 (s, 1H, OH-9), 4.45 (q, 1H, *J* = 12.4 Hz, H_2_-20), 3.29 (br s, 1H, H-10), 3.00 (t, 1H, *J* = 5.4 Hz, H-8), 2.55 (sex, *J* = 6.4 Hz, 1H, COCH(CH_3_)_2_), 2.49 (d, 1H, *J* = 18.9 Hz, H-5α), 2.38 (d, 1H, *J* = 18.9 Hz, H-5β), 2.23 (br s, 1H, OH-4), 2.08 (1H, overlapped, H-12α), 2.05 (s, 3H, CH_3_CO), 1.99–1.93 (m, 1H, H-11), 1.79 (br s, 3H, H_3_-19), 1.53 (dd, 1H, *J* = 14.4, 11.1 Hz, H-12β), 1.25 (s, 3H, H_3_-16), 1.16 (d, 6H, *J* = 6.4Hz, COCH(CH_3_)_2_), 1.08 (s, 3H, H-17), 0.88 (d, 3H, *J* = 6.4 Hz, H_3_-18), 0.80 (d, H, *J* = 5.4 Hz, H-14).^13^C NMR (101 MHz, CDCl_3_, δ ppm): δ = 209.3, 179.2, 171.0, 161.6, 134.9, 134.1, 132.9, 76.1, 73.7, 69.9, 63.2, 55.8, 39.6, 39.1, 36.5, 34.4, 32.6, 31.9, 23.4, 23.0, 21.1, 18.9, 18.8, 18.7,15.5, 10.3. HRMS-ESI (*m*/*z*): calcd for C_26_H_36_O_7_[M-H]^−^ 459.2388; found 459.2395, t_R_ = 5.63 min.

#### 4.4.3. 7-*p*-Metoxyphenylacetate-3,8,12-triacetate ingol (**3**)

White, amorphous powder; R*_f_*= 0.25 (20% EtOAc/hexane); m.p. 149–150 °C; ^1^H NMR (400 MHz, CDCl_3_, δ ppm, *J* Hz): δ = 7.18 (d, 2H, *J* = 8.7 Hz, aromatic), 6.85 (d, 2H, *J* = 8.7 Hz, aromatic), 5.40 (br s, 1H, H-5), 5.16 (d, 1H, *J* = 8.5 Hz, H-3), 5.13 (d, 1H, *J* = 1.8 Hz, H-7), 4.83 (dd, 1H, *J* = 10.8, 3.9 Hz, H-12), 4.53 (dd, 1H, *J* = 10.5, 1.8 Hz, H-8), 3.79 (s, 3H, OMe), 3.65 (s, 2H, COCH_2_Ar), 2.91–2.85 (m, 1H, H-13), 2.77 (dd, 1H, *J* = 14.9, 9.0 Hz, H-1α), 2.53–2.47 (m, 1H, H-2), 2.09 (s, 3H, H_3_-3OAc), 2.06 (s, 3H, H_3_-12 OAc), 2.06 (d, 3H, *J* = 1.2 Hz, H_3_-17), 1.97 (s, 3H, H_3_-8 OAc), 1.67 (d, 1H, *J* = 14.9 Hz, H-1β), 1.10 (1H, overlapped, H-9), 1.06 (1H, overlapped, H-11), 1.05 (s, 3H, H_3_-18), 1.02 (d, 3H, *J* = 7.3 Hz, H_3_-20), 0.92 (d, 3H, *J* = 7.5 Hz, H_3_-16), 0.83 (s, 3H, H_3_-19). ^13^C NMR (101 MHz, CDCl_3_, δ ppm): δ = 207.7, 170.8, 170.7, 170.5, 170.4, 158.9, 139.5, 130.4 (2 × C), 126.0, 117.2, 114.2 (2 × C), 77.0, 76.9, 73.4, 71.8, 71.2, 70.8, 55.2, 43.2, 40.7, 31.6, 31.0, 29.6, 29.2, 24.8, 21.1, 21.0, 20.7, 19.4, 17.6, 17.0, 16.2, 13.5. HRMS-ESI (*m*/*z*): calcd for C_35_H_44_O_11_ [M-H]^−^ 639.2811; found 639.2839, t_R_ = 6.39 min.

#### 4.4.4. Resiniferatoxin (**4**)

White powder; R*_f_* = 0.40 (32% EtOAc/hexane); m.p. 65–66 °C; ^1^H NMR (400 MHz, CDCl_3_, δ ppm, *J* Hz): δ = 7.44 (br s, 1H, H-1), 7.38–7.36 (m, 2H, aromatic), 7.30–7.20 (m, 3H, aromatic), 6.83 (d, 1H, *J* = 8.0 Hz, aromatic), 6.80–6.74 (m, 2H, aromatic), 5.87 (br s, 1H, H-7), 5.58 (br s, 1H, OH), 4.71 (br s, 2H, H-17), 4.60 (d, 1H, *J* = 12.2 Hz, H-20β), 4.53 (d, 1H, *J* = 12.2 Hz, H-20α) 4.20 (d, 1H, *J* = 2.7 Hz, H-14), 3.88 (s, 3H, OMe), 3.55 (s, 2H, COCH_2_Ar), 3.21 (s, 2H, CCH_2_Ph), 3.08 (br s, 1H, H-8), 3.04 (br s, 1H, H-10), 2.55 (dq, 1H, *J* = 8.0, 7.1 Hz, H-11), 2.43 (d, 1H, *J* = 18.7 Hz, H-5β), 2.13 (dd, 1H, *J* = 14.3, 8.7 Hz, H-12β), 2.06 (s, 1H, OH-4), 2.04 (d, 1H, *J* = 18.7 Hz, H-5α), 1.82–1.81 (m, 3H, H_3_-19), 1.56 (d, 1H, *J* = 14.3 Hz, H-12α),1.52 (s, 3H, H_3_-16), 0.96 (d, 3H, *J* = 7.1 Hz, H_3_-18). ^13^C NMR (101 MHz, CDCl_3_, δ ppm): δ = 208.6, 171.6, 158.4, 146.6, 146.5, 145.0, 136.7, 135.1, 134.2, 130.9 (2 × C), 128.7, 127.8 (2 × C), 126.6, 125.8, 122.3, 117.9, 114.5, 111.9, 110.9, 84.6, 81.2, 80.8, 73.4, 70.6, 56.1, 55.5, 41.2, 41.1, 40.1, 39.2, 35.8, 33.2, 20.0, 18.9, 10.4. HRMS-ESI (*m*/*z*): calcd for C_37_H_40_O_9_[M + H]^+^ 629.2745; found 629.2739, t_R_ = 6.39 min.

#### 4.4.5. Deglucosyl Euphorbioside A (**5**)

White, amorphous powder; R*_f_* = 0.25 (20% MeOH/EtOAc); m.p. 94–95 °C; ^1^H NMR (400 MHz, CD_3_OD, δ ppm, *J* Hz): δ = 5.80 (dd, 1H, *J* = 15.3, 5.8 Hz, H-8), 5.69 (dd, 1H, *J* = 15.3, 9.6 Hz, H-7), 4.32 (q, 1H, *J* = 6.4 Hz, H-9), 4.02 (d, 1H, *J* = 8.0 Hz, H-11β), 3.74 (ddd, 1H, *J* = 10.4, 7.8, 6.5 Hz, H-3), 3.55 (d, 1H, *J* = 7.8 Hz, H-2), 3.35 (1H, overlapped, H-11α), 2.19 (d, 1H, *J* = 9.6 Hz, H-6), 1.94 (dd, 1H, *J* = 13.6, 6.5 Hz, H-4β), 1.66 (dd, 1H, *J* = 13.6, 10.4 Hz, H-4α), 1.29 (d, 3H, *J* = 6.4 Hz, H_3_-10), 1.18 (s, 3H, H_3_-13), 1.04 (s, 3H, H-12). ^13^C NMR (101 MHz, CD_3_OD, δ ppm): δ = 142.4 (C-8), 123.7 (C-7), 83.8 (C-5), 76.6 (C-2), 74.1 (C-11), 73.1 (C-3), 68.9 (C-9), 61.3 (C-6), 49.3 (C-1), 42.9 (C-4), 24.5 (C-10), 23.9 (C-13), 18.0 (C-12). HRMS-ESI (*m*/*z*): calcd for C_13_H_22_O_4_ [M + H-H_2_O]^+^ 225.1485; found 225.1481, t_R_ = 2.32 min.

#### 4.4.6. Euphorbioside A (**6**)

White, amorphous powder; R*_f_* = 0.44 (40% MeOH/EtOAc); m.p. ˃ 300 °C; ^1^H NMR (400 MHz, CD_3_OD, δ ppm, *J* Hz): 5.81 (dd, 1H, *J* = 15.3, 5.8 Hz, H-8), 5.67 (dd, 1H, *J* = 15.9, 9.6 Hz, H-7), 4.35 (d, 1H, *J* = 7.8 Hz, H-1′), 4.35 (1H, overlapped, H-9), 4.08 (d, 1H, *J* = 8.0 Hz, H-11β), 3.94 (dd, 1H, *J* = 11.8, 1.8 Hz, H-6′α), 3.89 (ddd, 1H, *J* = 10.4, 7.8, 6.5 Hz, H-3), 3.70 (dd, 1H, *J* = 11.8, 6.1 Hz, H-6′β), 3.58 (d, 1H, *J* = 7.8 Hz, H-2), 3.39 (3H, overlapped, H-3′, H-5′ and H-11α), 3.35 (dd, 1H, *J* = 8.4, 7.1 Hz, H-4′), 3.30 (t, 1H, *J* = 7.8 Hz, H-2′), 2.20 (d, 1H, *J* = 9.6 Hz, H-6), 2.0 (dd, 1H, *J* = 13.6, 6.5 Hz, H-4β), 1.70 (dd, 1H, *J* = 13.6, 10.4 Hz, H-4α), 1.30 (d, 3H, *J* = 6.4 Hz, H_3_-10), 1.20 (s, 3H, H_3_-13), 1.16 (s, 3H, H_3_-12). ^13^C NMR (101 MHz, CD_3_OD, δ ppm): δ = 142.7 (C-8), 123.5 (C-7), 105.4 (C-1′), 88.1 (C-5′), 83.5 (C-5), 77.9 (C-2 and C-2′), 75.3 (C-3′), 74.6 (C-11), 71.8 (C-3), 71.5 (C-4′), 68.9 (C-9), 62.5 (C-6′), 61.2 (C-6), 49.8 (C-1), 42.0 (C-4), 24.3 (C-10), 23.9 (C-13), 18.2 (C-12). HRMS-ESI (*m*/*z*): calcd for C_19_H_32_O_9_ [M + Na]^+^ 427.1939; found 427.1928, t_R_ = 2.18 min.

#### 4.4.7. Catechol (**7**)

Light green solid. R*_f_* = 0.36 (30% EtOAc/hexane); m.p. 243–244 °C; ^1^H NMR (400 MHz, CD_3_OD, δ ppm, *J* Hz): 6.71 (dd, 2H, *J* = 5.9, 3.5 Hz, Ph-H), 6.61 (dd, 2H, *J* = 5.9, 3.5 Hz, Ph-H). ^13^C NMR (101 MHz, CD_3_OD, δ ppm): δ = 146.3 (2 × C), 120.9 (2 × CH), 116.4 (2 × CH). HRMS-ESI (*m*/*z*): calcd for C_6_H_6_O_2_ [M-H]^−^ 109.0295; found 109.0277, t_R_= 1.80 min.

#### 4.4.8. Protocatechuic Acid (**8**)

Yellow powder. R*_f_* = 0.59 (10% MeOH/EtOAc); m.p. 198–199 °C. ^1^H NMR (400 MHz, CD_3_OD, δ ppm, *J* Hz): 7.41–7.39 (m, 2H, Ph-H), 6.77 (d, 1H, *J* = 8.5 Hz, Ph-H). ^13^C NMR (101 MHz, CD_3_OD, δ ppm): δ = 170.3 (CO), 151.5 (C), 146.0 (C), 123.9 (CH), 123.1 (C), 117.7 (CH), 115.7 (CH). HRMS-ESI (*m*/*z*): calcd for C_7_H_6_O_4_ [M-H]^−^ 153.0193; found 153.0178, t_R_ = 1.50 min.

#### 4.4.9. 3,4-Dihydroxyphenylacetic Acid (**9**)

White, amorphous powder; R*_f_* = 0.26 (10% MeOH/EtOAc); m.p. 125–126 °C. ^1^H NMR (400 MHz, CD_3_OD, δ ppm, *J* Hz): 6.55 (d, 1H, *J* = 8.5 Hz, Ph-H), 6.51 (d, 1H, *J* = 2.8 Hz, Ph-H), 6.45 (dd, 1H, *J* = 8.5, 2.8 Hz, Ph-H), 3.42 (2H, S, CH_2_). ^13^C NMR (101 MHz, CD_3_OD, δ ppm): δ = 177.4 (CO), 151.1 (C), 149.7 (C), 124.3 (C), 118.4 (CH), 117.2 (CH), 115.4 (CH), 38.5 (CH_2_). HRMS-ESI (*m*/*z*): calcd for C_8_H_8_O_4_ [M-H]^−^ 167.0350; found 167.0340, t_R_ = 1.00 min.

The ^1^H NMR, ^13^C NMR, and LC/HRMS spectra for compounds **1**–**9** are available in the Supplementary Data.

### 4.5. Fungal Culture

The yeast strain that was used in this study was *Saccharomyces cerevisiae* strain W303 (MATa leu2–3112, trp1–1, can1–100, ura3–1, ade2–1, his3–11,15). A pre-culture of the yeast strain was grown in YPD, a culture-rich medium (1% bactopeptone, 1% yeast extract, and 2% glucose) for 24 h, then tubes with fresh medium were inoculated from the pre-culture (2 mL of YPD liquid medium) in which the compounds were added at the final concentration of 400 µM. The cultures were grown overnight. The cells were then counted, and serial dilutions (1 × 10^7^, 1 × 10^6^, 1 × 10^5^, 1 × 10^4^, 1 × 10^3^/mL) were spotted on YPD plates and grown at 28 °C for 24 h. To monitor the intra-cellular ROS levels, we used dihydrorodamine123, a dye that reacts with ROS to generate a fluorophore. The ROS levels were detected in exponential phase cultures that were treated with the compounds. Cell visualization was performed using the Axio Observer (ApoTome-Zeiss, Oberkochen, Germany). H_2_O_2_ sensitivity was tested plating the treated cells on YPD plates and spotting 5 μL of H_2_O_2_ 9,2 M on a sterile paper disk. After 24 h, the diameter of the halo was measured.

A black Aspergillus isolate (*A. carbonarius*) from wine grapes that were grown in the countryside of Manduria (Taranto, Italy) was used. The isolate was unambiguously identified by molecular techniques and maintained in CDA (Czapek-Dox Agar, Difco-BD, Franklin Lakes, N J, USA) slants at 25 °C.

### 4.6. Antifungal Assay

*Aspergillus carbonarius* conidia from a culture tube that were incubated for 10 days at 25 °C were scraped off and put in sterile distilled water. The conidia suspension was counted by a Thoma counting chamber (Merck, Darmstadt, Germany) and diluted with sterile distilled water to 3 × 10^5^ in 0.1 mL. That quantity was introduced into each one of the 50 mL-Erlenmeyer flasks that was filled with 25 mL of Czapek-Dox-Yeast 0.2% (CDA + Yeast extract 0.2%), a medium conducive for *A. carbonarius* growth. Each compound was dissolved in DMSO, and 50 µL of solutions subsequently diluted to obtain 10 µM, 50 µM, and 100 µM in the Erlenmeyer flask were added at the same time as the fungal inoculum. The fungal growth was evaluated after 3, 6, 11, and 16 days of incubation at 25 °C, by weighing the mycelial part of the cultures, that were previously filtered through filter paper on a separatory funnel, and dried at 80 °C for 48 h.

### 4.7. Cell Culture

Cell lines MCF7 and MCF10A were provided by the American Type Culture Collection (ATCC, Manassas, VA, USA). Human breast cancer cells MCF7 were cultured in RPMI 1640 medium containing 10% (*v*/*v*) fetal bovine serum (Invitrogen, Waltham, MA, USA), The MCF10A normal breast cells were cultured in Dulbecco’s modified Eagle’s medium and F12 medium (DMEM-F12) which was supplemented with horse serum (5%), hydrocortisone (0.5 μg/mL), EGF (20 ng/mL), and insulin (10 μg/mL), [46,47]. The maintained conditions were provided at 37 °C with an atmosphere of 5% CO_2_ and 100% humidity. The statistical significance was calculated by comparing the two groups: MCF7 and MCF10A cells that were treated at 100 µM, and supports our conclusions.

### 4.8. Cytotoxicity Assay

Cells’ viability in the normal and treatment conditions was measured by the MTS assay (Promega, Wisconsin, IA, USA). In brief, the cells were cultured in adhesion conditions in TC-treated well at density 1000 cells/100 μL medium and treated with different compounds. After 24 h, the MTS solution was added per well and incubated at 37 °C for 3 h. Finally, the optical density (OD) was measured under the 492-nm wavelength, and the survival rates were calculated.

### 4.9. Mitochondrial ROS Formation in Human Cell Lines

Mitochondrial ROS formation was detected using MitoSOX™ Red (Thermo Fisher Scientific, Waltham, MA, USA), a highly selective indicator for the detection of superoxide in the mitochondria of living cells. Briefly, the cells were seeded in 96-well black plates at a density of 10 × 10^3^ cells per well and left to grow overnight. The cells were treated with the tested compounds for 1 h. The cells were then further incubated at 37 °C with 5 μΜ MitoSOX for 15 min in the dark. The fluorescence intensity of MitoSOX™ Red was recorded by using an Infinite M200 plate reader at an emission wavelength of 580 nm and at an excitation wavelength of 510 nm.

## 5. Conclusions

In conclusion, nine compounds were isolated from the dried *E. resinifera* latex. Among these, catechol (**7**), protocatechuic acid (**8**), and 3,4-dihydroxyphenylacetic acid (**9**), known phenolic compounds, were identified for the first time in *E. resinifera* latex. Other known compounds were also identified (**1**–**6**). Extensive NMR and mass spectroscopic analysis were used to elucidate their structures. Most of the isolated compounds were tested for their antifungal and cytotoxic activities. We demonstrated that 12-deoxyphorbol-13-isobutyrate-20-acetate (**2**) had an inhibiting effect on the growth of *A. carbonarius,* and 7-*p*-metoxyphenylacetate-3,8,12-triacetate ingol (**3**) showed a cytotoxic effect on breast cancer cell line MCF7. Furthermore, deglucosyl euphorbioside A (**5**) and euphorbioside A (**6**) showed a discoloration effect that was possibly related to mitochondrial functionality in yeast, and compound **5** also showed a specific cytotoxic effect on the cancer cell lines MCF7. The results suggest that *E. resinifera* latex may contain compounds with control activity against cancerous cells and contaminant molds. Further investigations will be carried out on the most active compounds in order to study their mechanisms of action.

## Data Availability

Not applicable.

## Supplementary Materials

The following supporting information can be downloaded at: https://www.mdpi.com/article/10.3390/molecules27165234/s1, The ^1^H NMR, ^13^C NMR, and LC/HRMS spectra for compounds **1**–**9** are available in the Supplementary Data.

## Sample Availability

Samples of the compounds are available from the authors.

## References

[1] Bray F., Ferlay J., Soerjomataram I., Siegel R.I., Torre L.A., Jemal A. (2018). Global cancer statistics 2018: GLOBOCAN estimates of incidence and mortality worldwide for 36 cancers in 185 countries. CA Cancer J. Clin..

[2] Torres H.A., Rivero G.A., Lewis R.E., Hachem R., Raad I., Kontoyiannis D.P. (2003). Aspergillosis caused by non-*fumigatus Aspergillus* species: Risk factors and in vitro susceptibility compared with *Aspergillus fumigatus*. Diagn. Microbiol. Infect. Dis..

[3] Bérubé G. (2019). How to utilize academic research efforts in cancer drug discovery. Expert Opin. Drug Discov..

[4] Nicola A.M., Albuquerque P., Paes H.C., Fernandes L., Costa F.F., Kioshima E.S., Abadio A.K.R., Bocca A.L., Felipe M.S. (2018). Antifungal drugs: New insights in research & development. Pharmacol. Ther..

[5] Ernst M., Saslis-Lagoudakis C.H., Grace O.M., Nilsson N., Toft Simonsen H., Horn J.W., Rønsted N. (2016). Evolutionary prediction of medicinal properties in the genus *Euphorbia* L.. Sci. Rep..

[6] Newman D.J., Cragg G.M. (2007). Natural products as sources of new drugs over the last 25 years. J. Nat. Prod..

[7] Singla A., Pathak K. (1990). Phytoconstituents of *Euphorbia* species. Fitoterapia.

[8] Fattorusso E., Lanzotti V., Taglialatela-Scafati O., Tron G.C., Appendino G. (2002). Bisnorsesquiterpenoids from *Euphorbia resinifera* Berg. and an Expeditious Procedure to Obtain Resiniferatoxin from Its Fresh Latex. Eur. J. Org. Chem..

[9] Hergenhahn M., Kusumoto S., Hecker E. (1974). Diterpene esters from ‘Euphorbium’ and their irritant and cocarcinogenic activity. Experientia.

[10] Hergenhahn M., Adolf W., Hecker E. (1975). Resiniferatoxin and other esters of novel polyfuncticnal diterpenes from *Euphorbia resinifera* and *unispina*. Tetrahedron Lett..

[11] Zhao N.D., Ding X., Song Y., Yang D.Q., Yu H.L., Adelakun T.A., Qian W.D., Zhang Y., Di Y.T., Gao F. (2018). Identification of Ingol and Rhamnofolane Diterpenoids from *Euphorbia resinifera* and Their Abilities to Induce Lysosomal Biosynthesis. J. Nat. Prod..

[12] Wang S.Y., Li G.Y., Zhang K., Wang H.Y., Liang H.G., Huang C., Huang J., Wang J.H., Yang B.F. (2019). New ingol-type diterpenes from the latex of *Euphorbia resinifera*. J. Asian Nat. Prod. Res..

[13] Ponsinet G., Ourisson G. (1968). Aspects particuliers de la biosynthèse des triterpènes dans le latex d’*Euphorbia*. Phytochemistry.

[14] Mazoir N., Benharref A., Bailén M., Reina M., Gonzàlez-Coloma A. (2008). Bioactive triterpene derivatives from latex of two *Euphorbia* species. Phytochemistry.

[15] Wang S., Liang H., Zhao Y., Wang G., Yao H., Kasimu R., Wu Z., Li Y., Huang J., Wang J. (2016). New triterpenoids from the latex of *Euphorbia resinifera* Berg. Fitoterapia.

[16] Boe J.E., Winsnes R., Nordal A., Bernatek E. (1969). New constituents of *Euphorbia resinifera* Berg. Acta Chem. Scand..

[17] Fatope M.O., Zeng L., Ohayaga J.E., Shi G., McLaughlin J.L. (1996). Selectively Cytotoxic Diterpenes from *Euphorbia poisonii*. J. Med. Chem..

[18] Ragasa C.Y., Cornelio K.B. (2013). Triterpenes from *Euphorbia hirta* and their cytotoxicity. J. Nat. Med..

[19] Wang S.Y., Huang C., Sun R.K., Lu L.N., Liang H.G., Gao L., Huang J., Wang J.H., Yang B.F. (2019). New tirucallane triterpenoids from the dried latex of *Euphorbia resinifera*. Phytochem. Lett..

[20] Geribaldi-Doldán N., Flores-Giubi E., Murillo-Carretero M., García-Bernal F., Carrasco M., Macías-Sánchez A., Sánchez J., Domínguez-Riscart J., Verástegui C., Hernández-Galán R. (2015). 12-Deoxyphorbols Promote Adult Neurogenesis by Inducing Neural Progenitor Cell Proliferation via PKC Activation. Int. J. Neuropsychopharmacol..

[21] Kim C.H., Lim S.J., Gollapaudi S., Gupta S. (1994). Role of Protein Kinase C Isozymes in Activation of Human Immunodeficiency Virus Type 1 in Chronically Infected Promonocytic Cells: Evidence Against a Role of PKCβ1. Biochem. Biophys. Res. Commun..

[22] Daoubi M., Marquez N., Mazoir N., Benharref A., Hernández-Galán R., Munozc E., Isidro G., Colladoa I.G. (2007). Isolation of new phenylacetylingol derivatives that reactivate HIV-1 latency and a novel spirotriterpenoid from *Euphorbia officinarum* latex. Bioorg. Med. Chem..

[23] Farah H., Echchahad A., Lamiri A. (2014). Semi-synthesis and antimicrobial activities of some new euphorbioside derivatives. Int. J. Chem. Tech. Res..

[24] Mazoir N., Benharref A., Bailén M., Reina M., González-Coloma A., Martínez-Díaz R.A. (2011). Antileishmanial and antitrypanosomal activity of triterpene derivatives from latex of two *Euphorbia* species. Z. Nat. C J. Biosci..

[25] Vasas A., Hohmann J. (2014). *Euphorbia* diterpenes: Isolation, structure, biological activity, and synthesis (2008–2012). Chem. Rev..

[26] Wang H.B., Wang X.Y., Liu L.P., Qin G.W., Kang T.G. (2015). Tigliane Diterpenoids from the *Euphorbiaceae* and *Thymelaeaceae* Families. Chem. Rev..

[27] Hashimoto S., Katoh S.I., Kato T., Urabe D., Inoue M. (2017). Total Synthesis of Resiniferatoxin Enabled by Radical-Mediated Three-Component Coupling and 7-endo Cyclization. J. Am. Chem. Soc..

[28] Allred T.K., Manoni F., Harran P.G. (2017). Exploring the Boundaries of “Practical”: De Novo Syntheses of Complex Natural Product-Based Drug Candidates. Chem. Rev..

[29] Wender P.A., Jesudason C.D., Nakahira H., Tamura N., Tebbe A.L., Ueno Y. (1997). The First Synthesis of a Daphnane Diterpene: The Enantiocontrolled Total Synthesis of (+)-Resinifera toxin. J. Am. Chem. Soc..

[30] Cruz F., Guimaraes M., Silva C., Reis M. (1997). Suppression of bladder hyperreflexia by intravesical resinifera toxin. Lancet.

[31] Brederson J.D., Kym P.R., Szallasi A. (2013). Targeting TRP channels for pain relief. Eur. J. Pharmacol..

[32] Brown D.C. (2016). Resiniferatoxin: The Evolution of the “Molecular Scalpel” for Chronic Pain Relief. Pharmaceuticals.

[33] Szallazi A., Blumberg P.M. (1989). Resiniferatoxin, a phorbol-related diterpene, acts as an ultrapotent analog of capsaicin, the irritant constituent in red pepper. Neuroscience.

[34] Appendino G., Szallasi A. (1997). Euphorbium: Modern research on its active principle, resiniferatoxin, revives an ancient medicine. Life Sci..

[35] Popplewell W.L., Marais E.A., Brand L., Harvey B.H., Davies-Coleman M.T. (2010). Euphorbias of South Africa: Two New Phorbol Esters from *Euphorbia Bothae*. S. Afr. J. Chem..

[36] Adolf W., Sorg B., Hergenhahn M., Hecker E. (1982). Structure-activity relations of polyfunctional diterpenes of the daphnane type. I. revised structure for resiniferatoxin and structure-activity relations of resiniferonol and some of its esters. J. Nat. Prod..

[37] Huang S., Zhang C.P., Li G., Sun Y.Y., Wang K., Hu F.L. (2014). Identification of Catechol as a New Marker for Detecting Propolis Adulteration. Molecules.

[38] Gutzeit D., Wray V., Winterhalter P., Jerz G. (2007). Preparative Isolation and Purification of Flavonoids and Protocatechuic Acid from Sea Buckthorn Juice Concentrate (*Hippophae rhamnoides* L. ssp. *rhamnoides*) by High-Speed Counter-Current Chromatography. Chromatographia.

[39] Guazzaroni M., Crestini C., Saladino R. (2012). Layer-by-Layer coated tyrosinase: An efficient and selective synthesis of catechols. Bioorg. Med. Chem..

[40] Munro B., Vuong Q.V., Chalmers A.C., Goldsmith C.D., Bowyer M.C., Scarlett C.J. (2015). Phytochemical, Antioxidant and Anti-Cancer Properties of *Euphorbia tirucalli* Methanolic and Aqueous Extracts. Antioxidants.

[41] Cirigliano A., Stirpe A., Menta S., Mori M., Dell’Edera D., Pick E., Negri R., Botta B., Rinaldi T. (2016). Yeast as a tool to select inhibitors of the cullin deneddylating enzyme Csn5. J. Enzym. Inhib. Med. Chem..

[42] Ottaviano D., Montanari A., De Angelis L., Santomartino R., Visca A., Brambilla L., Rinaldi T., Bello C., Reverberi M., Bianchi M. (2015). Unsaturated fatty acids-dependent linkage between respiration and fermentation revealed by deletion of hypoxic regulatory KlMGA2 gene in the facultative anaerobe-respiratory yeast *Kluyveromyces lactis*. FEMS Yeast Res..

[43] Cirigliano A., Amelina A., Biferali B., Macone A., Mozzetta C., Bianchi M.M., Mori M., Botta B., Pick E., Negri R. (2019). Statins interfere with the attachment of *S. cerevisiae* mtDNA to the inner mitochondrial membrane. J. Enzym. Inhib. Med. Chem..

[44] Botta L., Filippi S., Zippilli C., Cesarini S., Bizzarri B.M., Cirigliano A., Rinaldi T., Paiardini A., Fiorucci D., Saladino R. (2020). Artemisinin Derivatives with Antimelanoma Activity Show Inhibitory Effect against Human DNA Topoisomerase 1. ACS Med. Chem. Lett..

[45] Cirigliano A., Macone M., Bianchi M.M., Oliaro Bosso S., Balliano G., Negri R., Rinaldi T. (2019). Ergosterol reduction impairs mitochondrial DNA maintenance in *S. cerevisiae*. BBA Mol. Cell Biol. Lip..

[46] Ashraf A., Sarfraz R., Rashid M., Shahid M. (2015). Antioxidant, antimicrobial, antitumor, and cytotoxic activities of an important medicinal plant (*Euphorbia royleana*) from Pakistan. J. Food Drug Anal..

[47] Van Deenen N., Prufer D., Gronover C. (2011). A latex lectin from *Euphorbia trigona* is a potent inhibitor of fungal growth. Biol. Plant..

[48] Sadeghi-Aliabadi H., Sajjadi S., Khodamoradi M. (2009). Cytotoxicity of *Euphorbia macroclada* on MDA-MB-468 breast cancer cell line. Iran. J. Pharm. Sci..

[49] Al-Faifi Z.I., Masrahi Y.S., Aly M.S., Al-Turki T.A., Dardeer T. (2017). Evaluation of Cytotoxic and Genotoxic Effects of *Euphorbia Triaculeata* Forssk. Extract. Asian Pac. J. Cancer Prev..

[50] Sarmiento-Salinas F.L., Delgado-Magallón A., Montes-Alvarado J.B., Ramírez-Ramírez D., Flores-Alonso J.C., Cortés-Hernández P., Reyes-Leyva J., Herrera-Camacho I., Anaya-Ruiz M., Pelayo R. (2019). Breast Cancer Subtypes Present a Differential Production of Reactive Oxygen Species (ROS) and Susceptibility to Antioxidant Treatment. Front. Oncol..

[51] Salimi V., Shahsavari Z., Safizadeh B., Hosseini A., Khademian N., Tavakoli-Yaraki M. (2017). Sodium butyrate promotes apoptosis in breast cancer cells through reactive oxygen species (ROS) formation and mitochondrial impairment. Lipids Health Dis..

