# Supplementary material for: Antifungal and Cytotoxic Activity of Diterpenes and Bisnorsesquiterpenoides from the Latex of Euphorbia resinifera Berg
Source: Molecules. 2022 Aug 16;27(16):5234. doi: 10.3390/molecules27165234 (PMC9413093; doi:10.3390/molecules27165234)

SUPPORTING INFORMATION

Antifungal and Cytotoxic Activity of Diterpenes and Bisnorsesquiterpenoides from the Latex of *Euphorbia resinifera* Berg

El-Mahdi Ourhzif [1,2,3], Alessandra Ricelli [4,*], Venturina Stagni [4], Angela Cirigliano [4], Teresa Rinaldi [5], Latifa Bouissane [3], Luciano Saso [6], Pierre Chalard [1], Yves Troin [1], Mostafa Khouili [3,*] and Mohamed Akssira [2,*]

1. Institut de Chimie de Clermont-Ferrand (ICCF), Dep. Chimie Organique et Medicinale, Université Clermont Auvergne CNRS SIGMA, F-63000 Clermont-Ferrand, France
2. Laboratoire de Chimie Physique et Biotechnologie des Biomolécules et des Matériaux, Faculté des Sciences et Techniques, Université Hassan II Casablanca, BP 146, Mohammedia 28800, Morocco
3. Laboratoire de Chimie Moléculaire, Matériaux et Catalyse (LCMMC), Faculté des Sciences et Techniques, Université Sultan Moulay Slimane, BP 523, Beni-Mellal 23000, Morocco
4. Institute of Molecular Biology and Pathology-CNR, P.le Aldo Moro, 5, 00185 Rome, Italy
5. Department of Biology and Biotechnology, "Charles Darwin" Sapienza University, P.le Aldo Moro, 5, 00185 Rome, Italy
6. Department of Physiology and Pharmacology, "Vittorio Erspamer" Sapienza University, P.le Aldo Moro, 5, 00185 Rome, Italy

* Correspondence: alessandra.ricelli@cnr.it (A.R.); m.khouili@usms.ma (M.K.); mohamed.akssira@fstm.ac.ma (M.A.)

Contents:

^{1}H and ^{13}C NMR spectra for all compounds **1-9** S2

LC/HRMS spectra for all products **1-9** S16

NMR (^{1}H, ^{13}C) SPECTRA:

12-Deoxyphorbol-13-angelate-20-acetate (1)

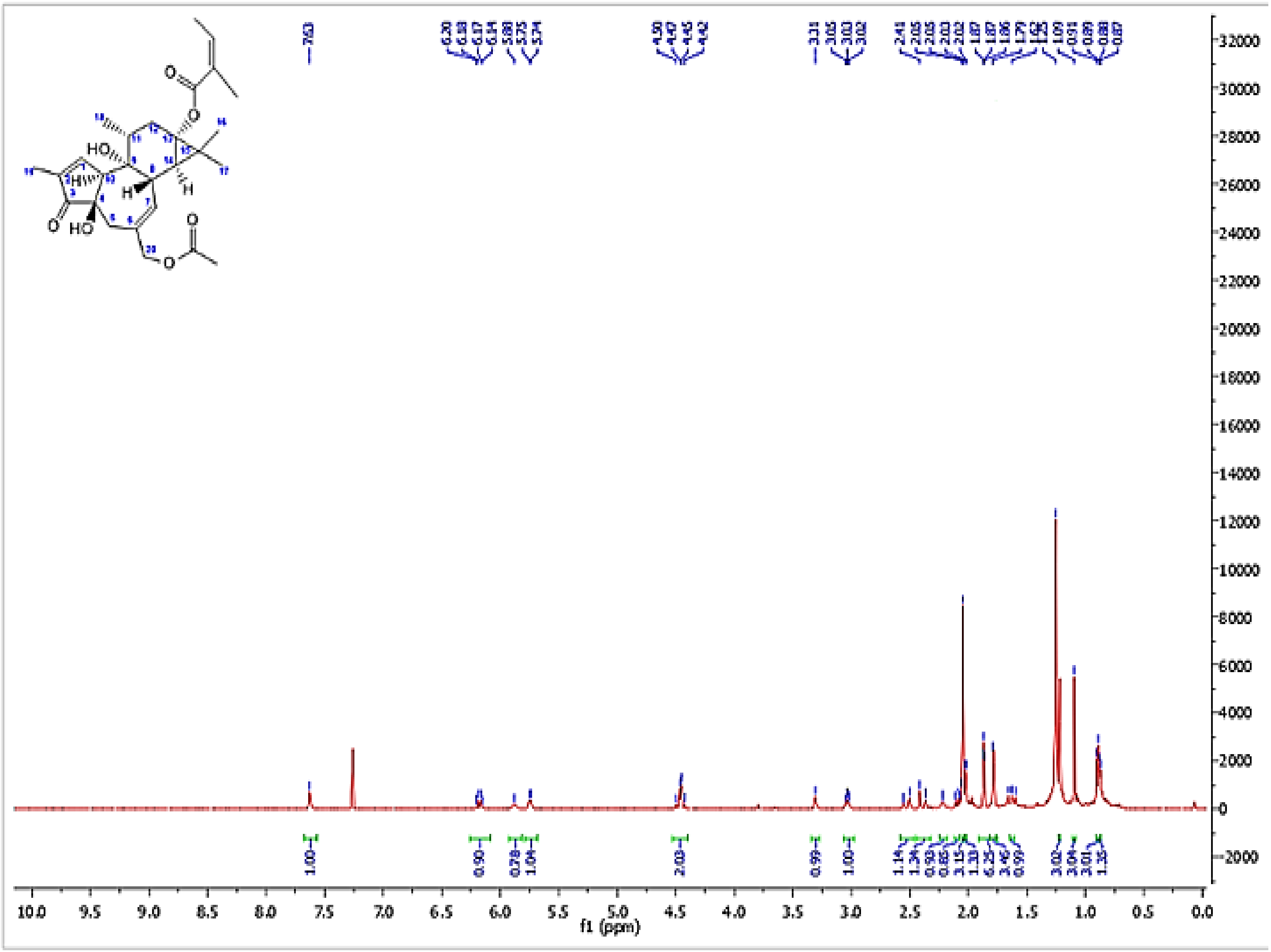

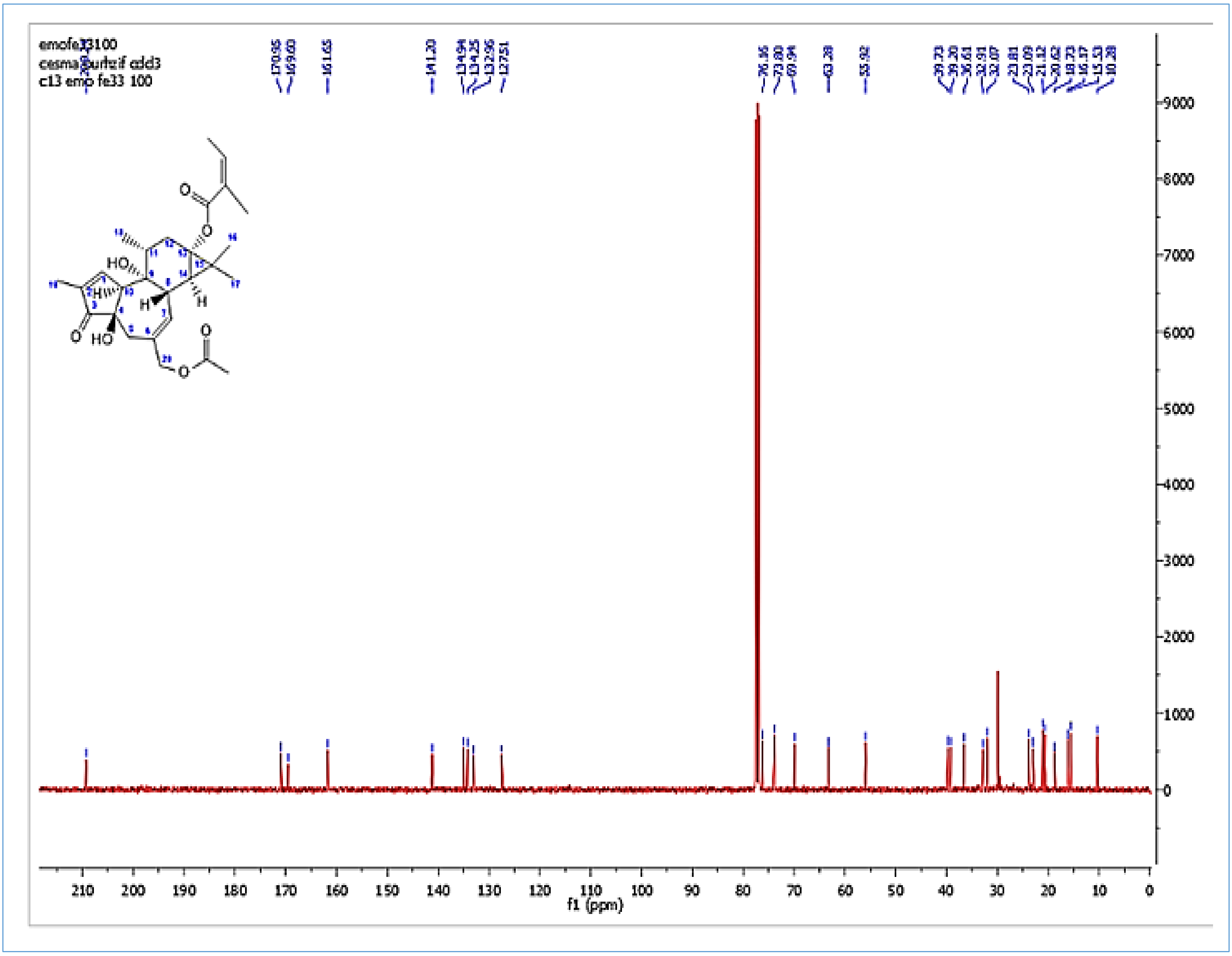
emofe33100
cesma@urfz if cdcl3
c13 emo fe33 100
170.95
169.03
161.65
141.20
134.54
134.25
132.96
127.51
76.56
73.80
69.94
63.28
55.92
39.73
39.20
36.61
32.91
32.07
23.81
23.09
21.12
20.62
18.73
16.17
15.53
10.28
f1 (ppm)

12-Deoxyphorbol-13-isobutyrate-20-acetate (2)

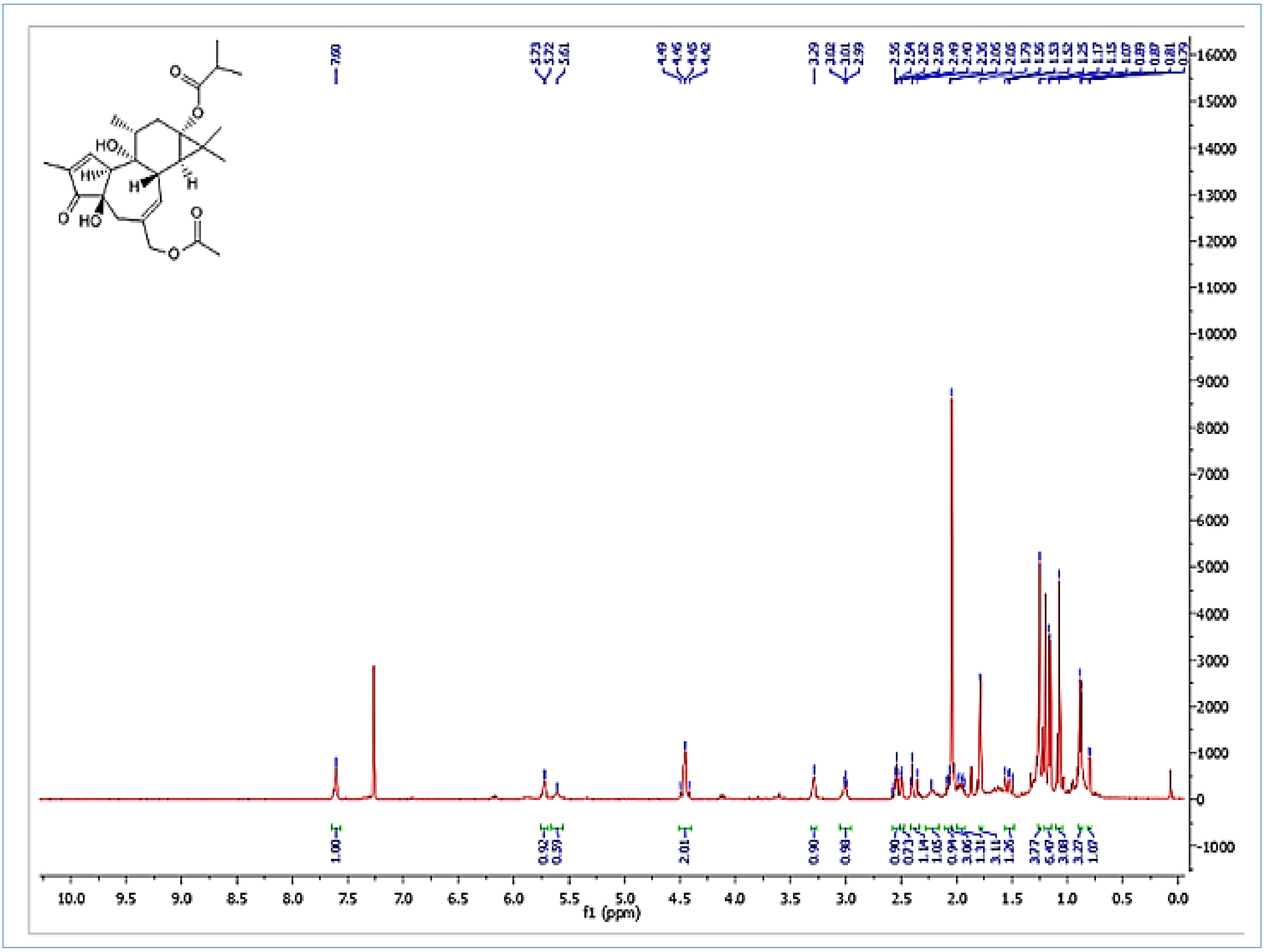

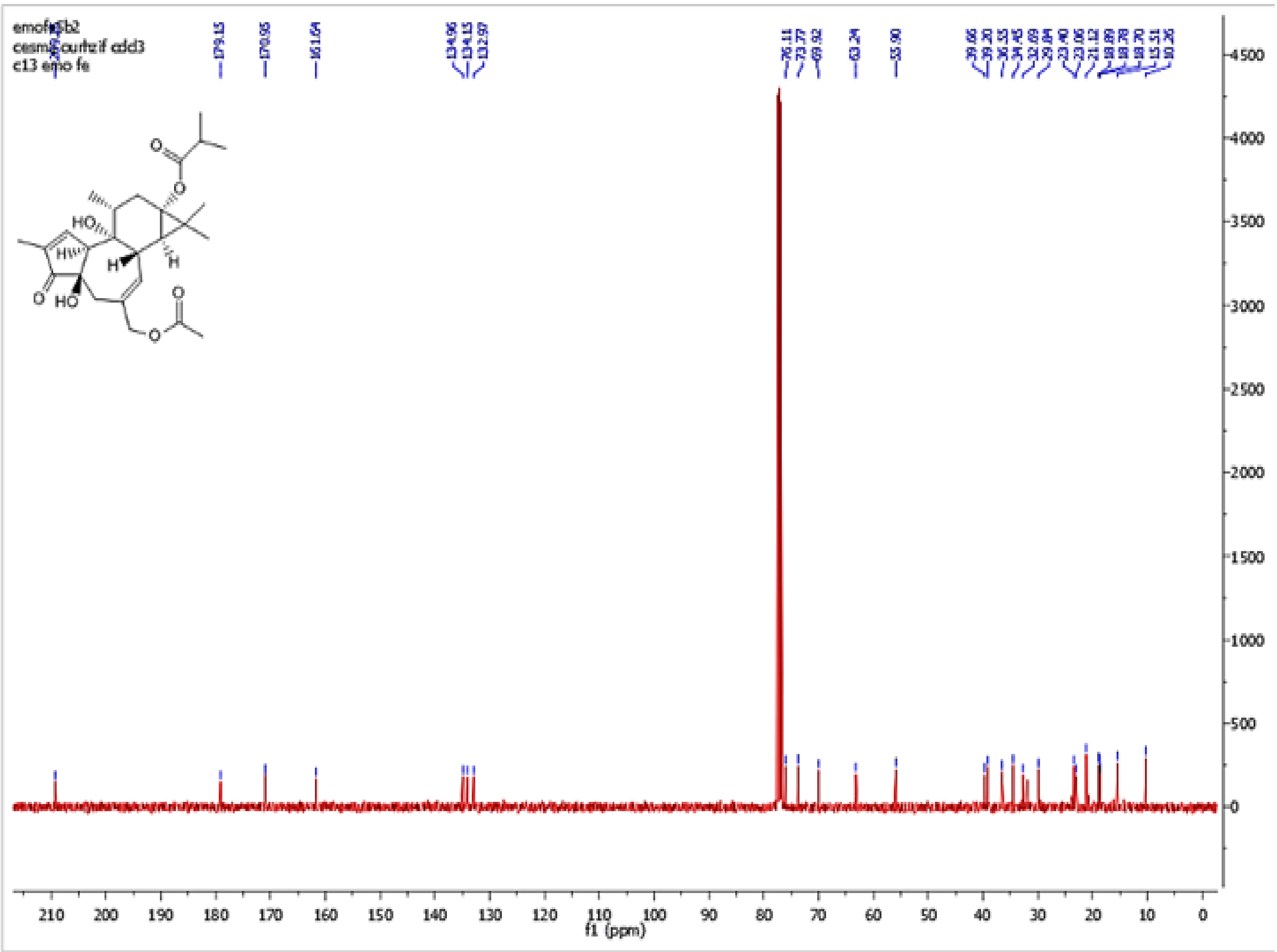

[13]C Attached Proton Test, CH_3/CH positive, CH_2/C negative (jmod)

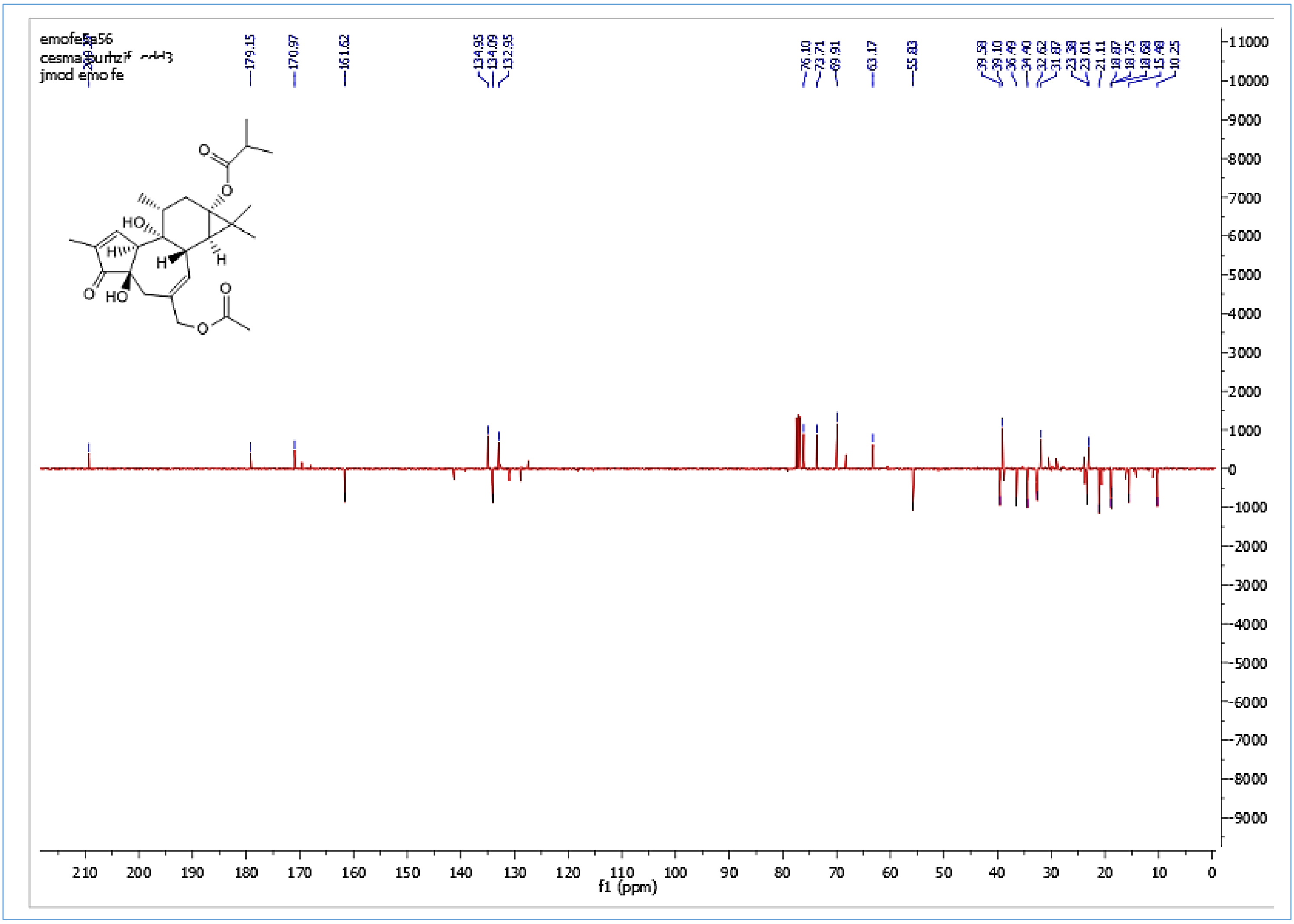

7-p-Métoxyphénylacétate-3,8,12-triacétate ingol (3)

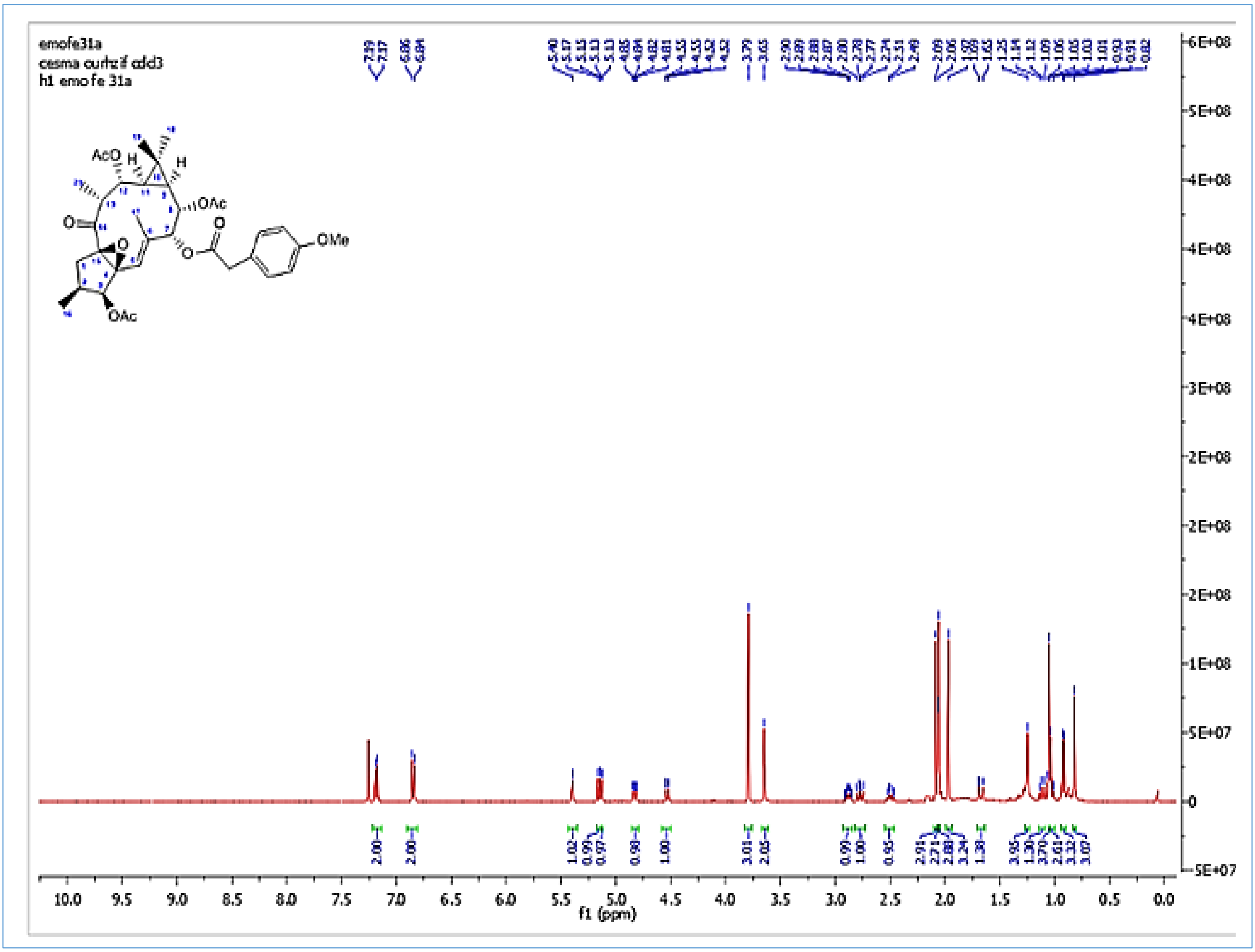

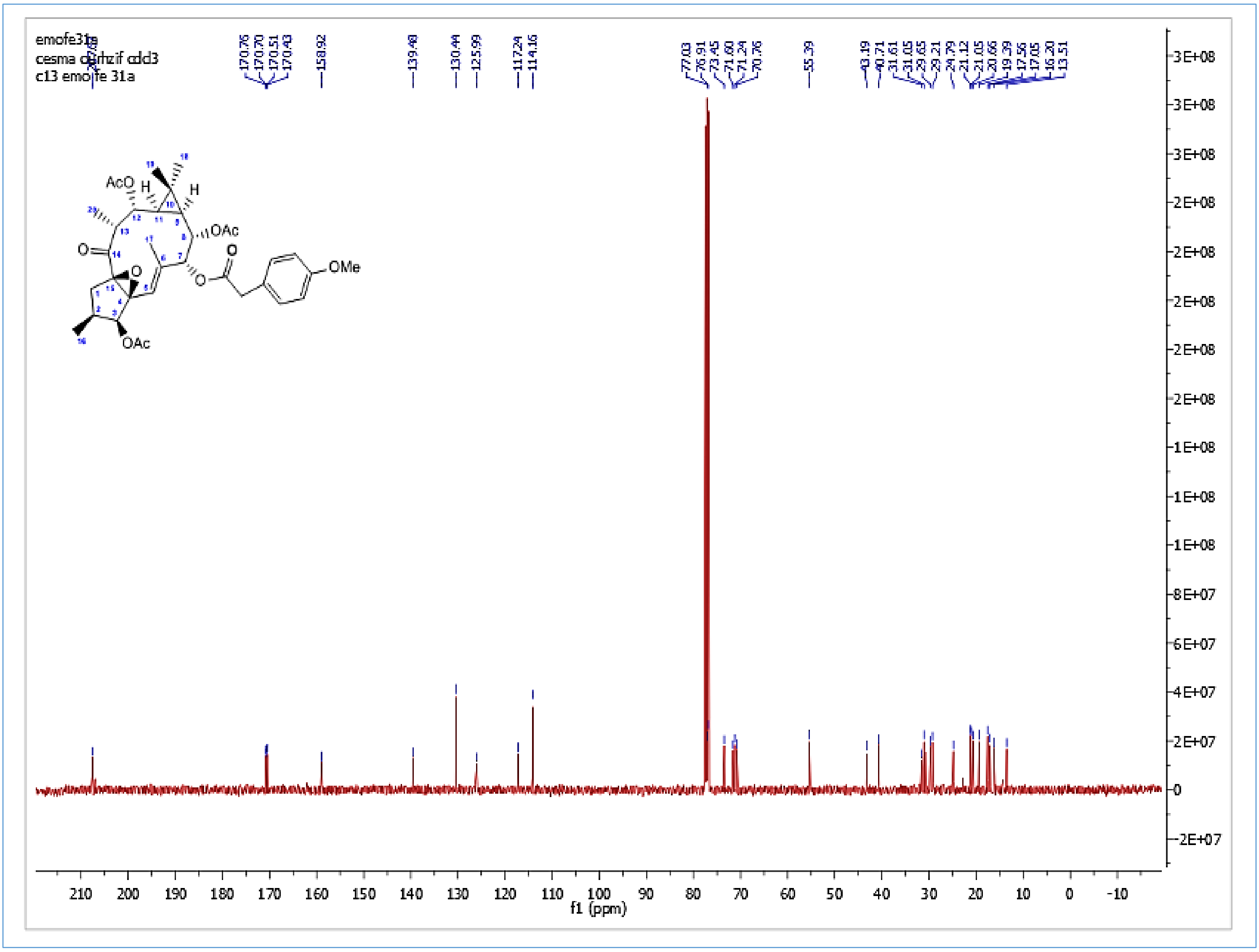

Resiniferatoxine (4)

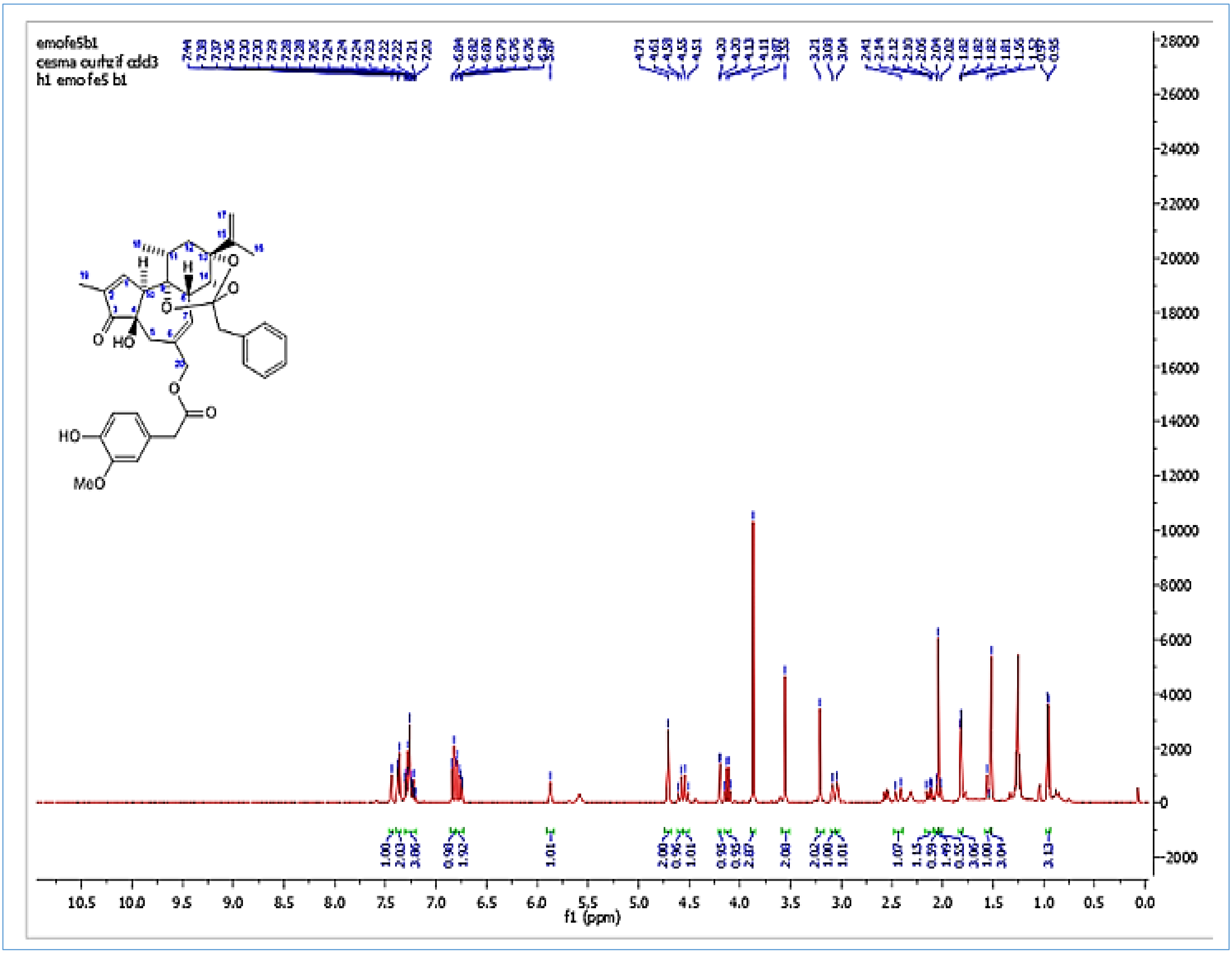

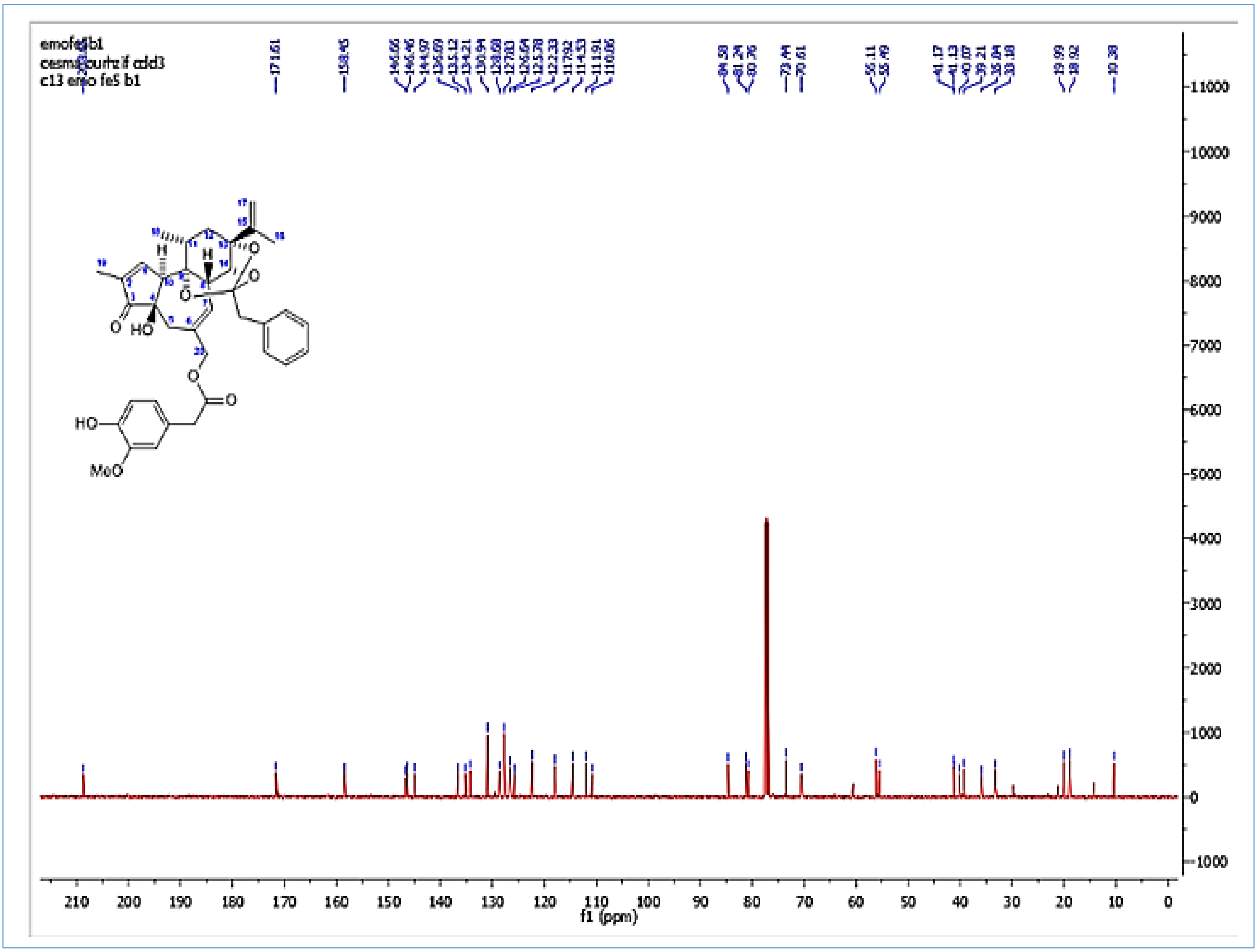
c13 emo fe5 b1
171.61
158.45
f1 (ppm)
HO
MeO
O
HO

Deglucosyl euphorbioside A (5)

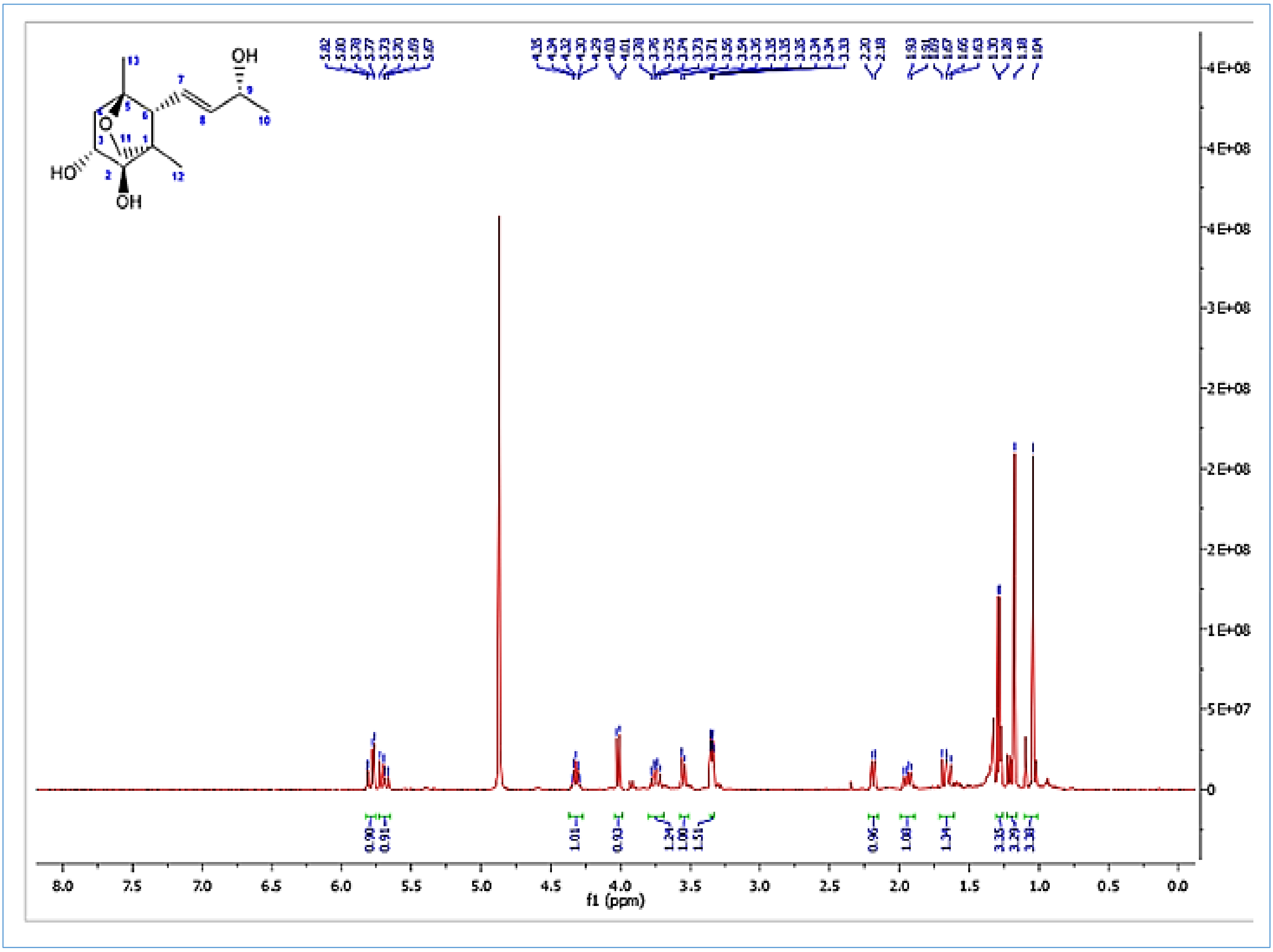

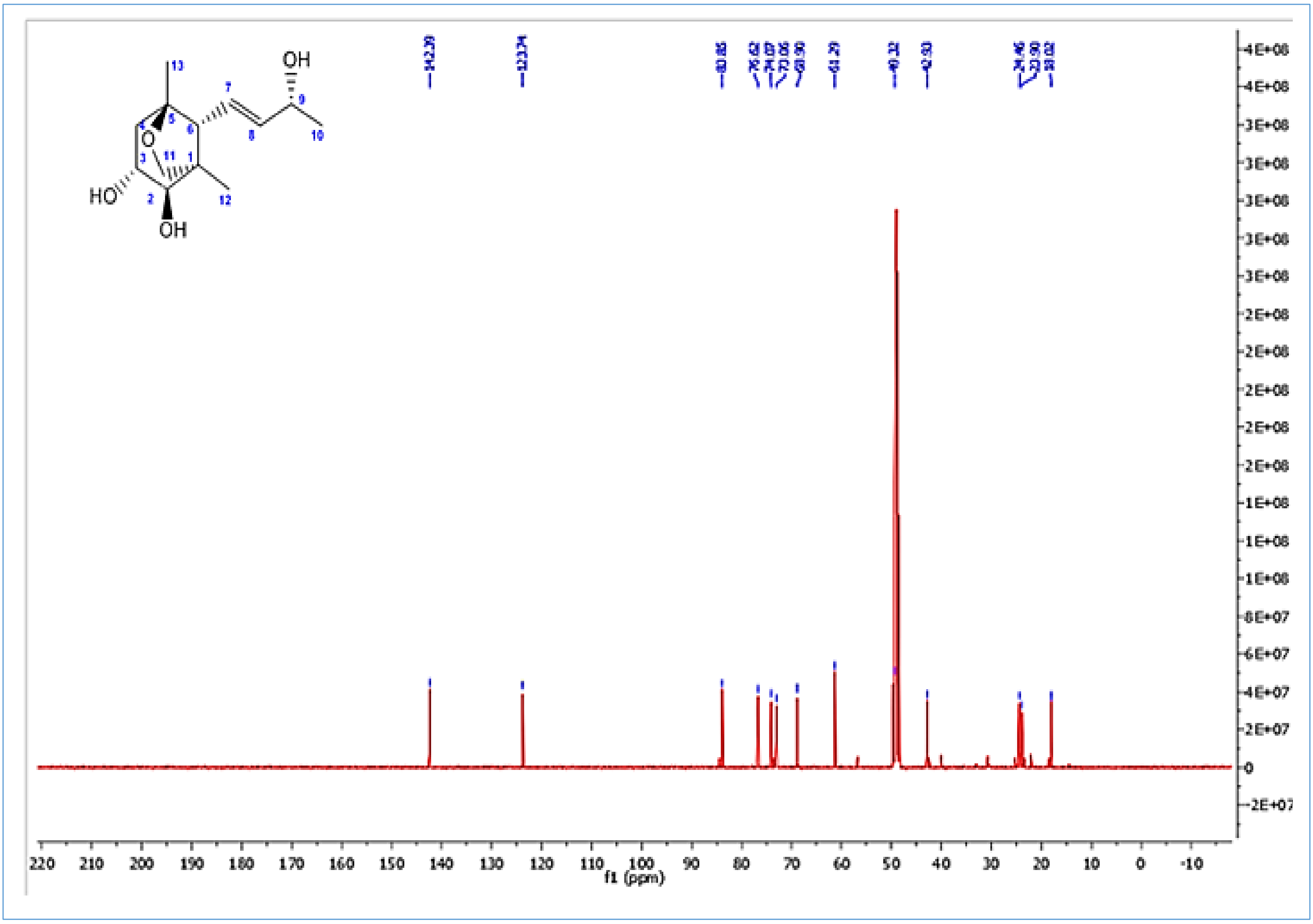
OH
HO
OH
O
f1 (ppm)

^{13}C Attached Proton Test, CH_3/CH positive, CH_2/C negative (jmod) and 2D HMQC

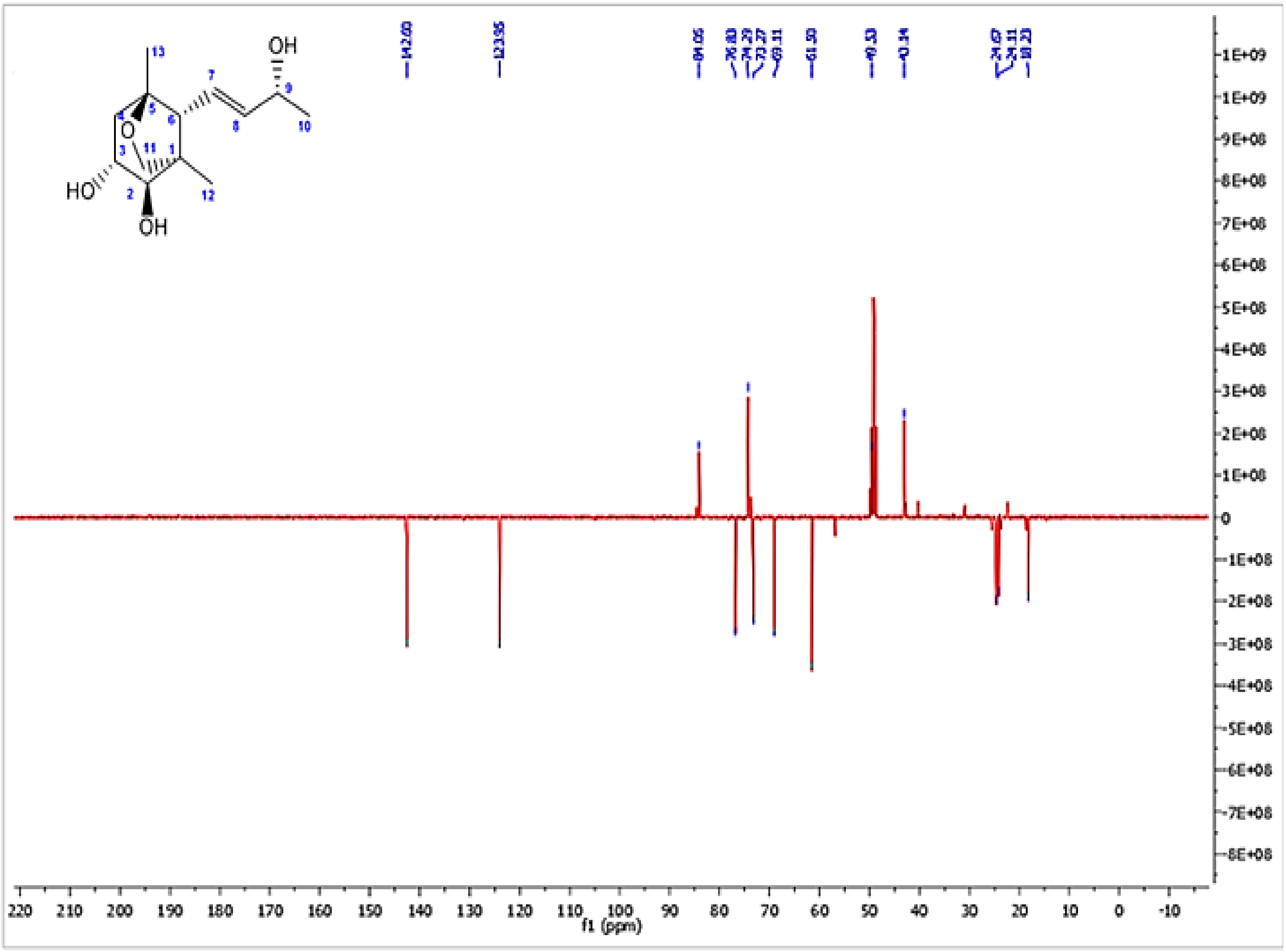

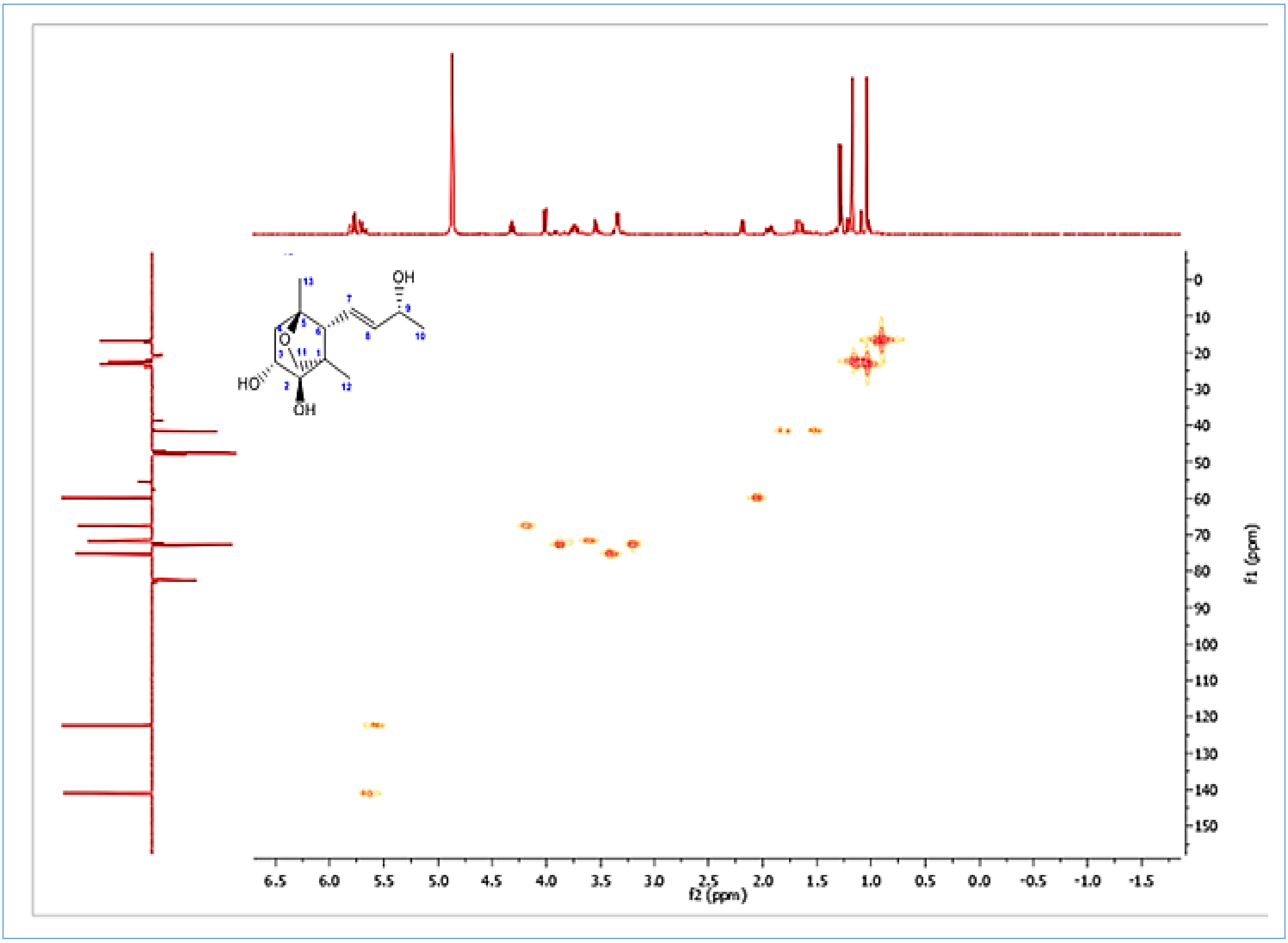
OH
HO
OH
O
f2 (ppm)
f1 (ppm)

Euphorbioside A (6)

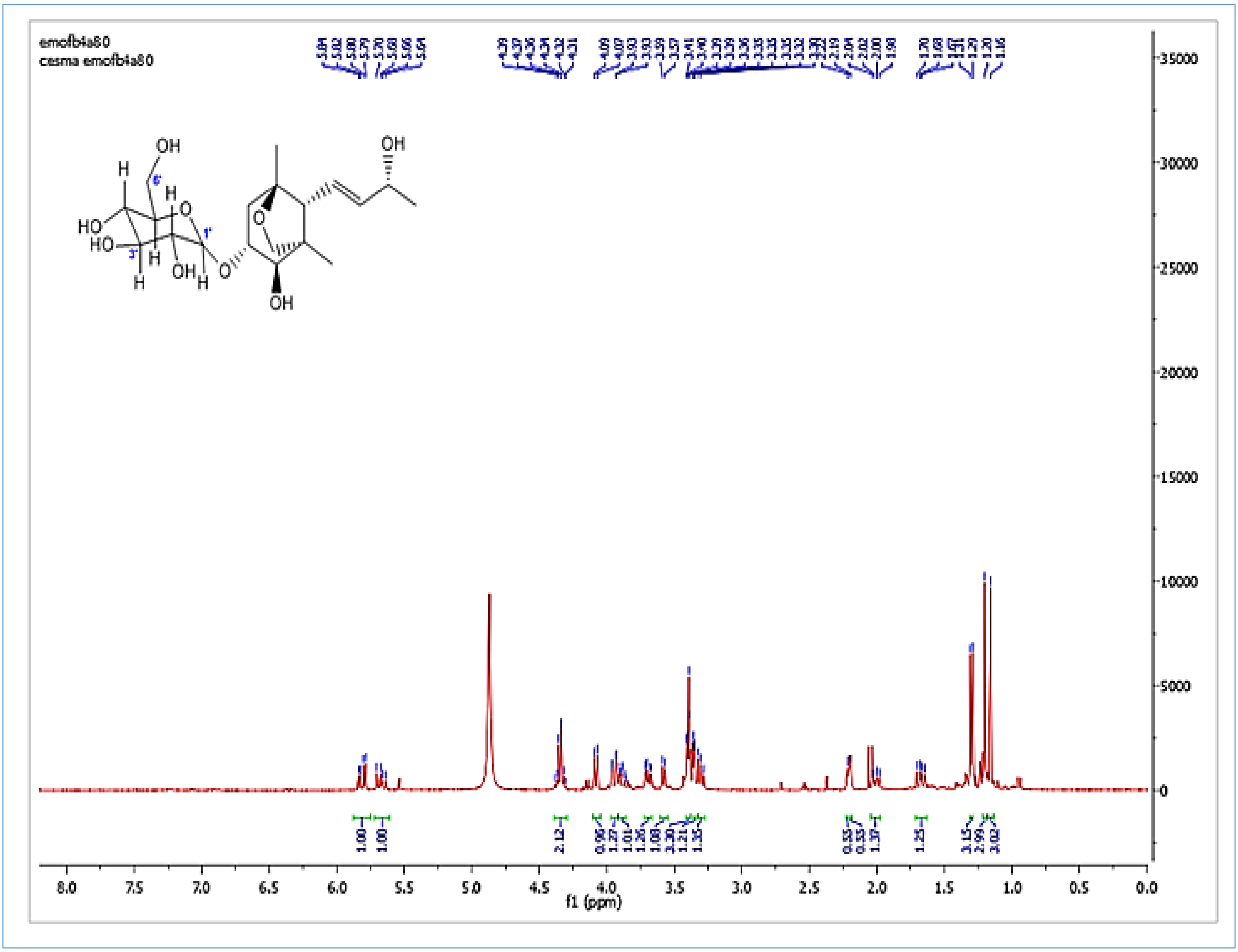

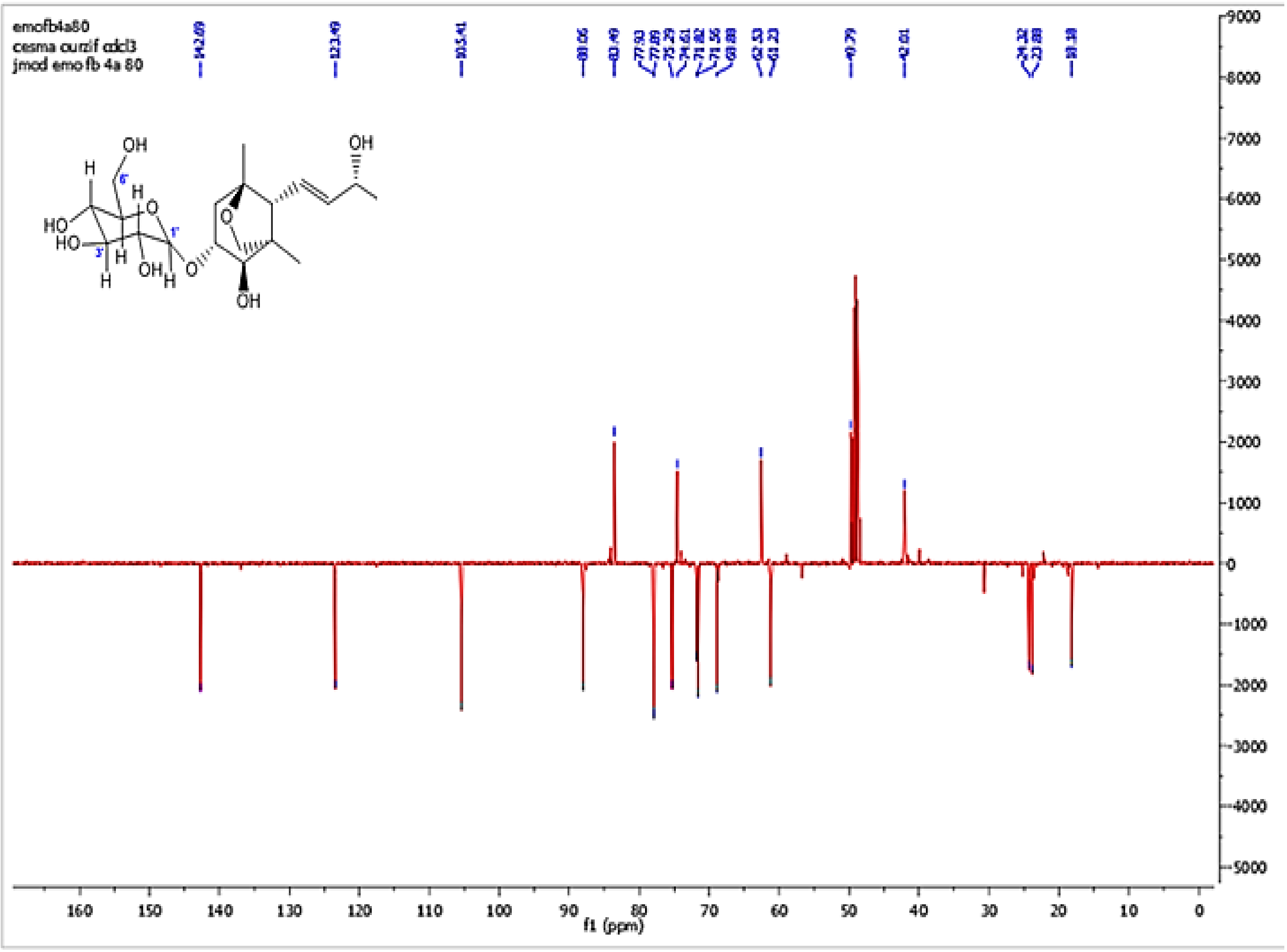

emofb4a80
cesma ourzif cdcl3
jmod emo fb 4a 80
142.09
123.49
105.41
88.06
83.49
77.93
77.89
75.29
74.61
71.82
71.56
68.88
62.53
61.23
49.79
42.01
24.32
23.88
18.18
OH
H
HO
HO
OH
O
H
6'
3'
1'
f1 (ppm)

Catechol (7)

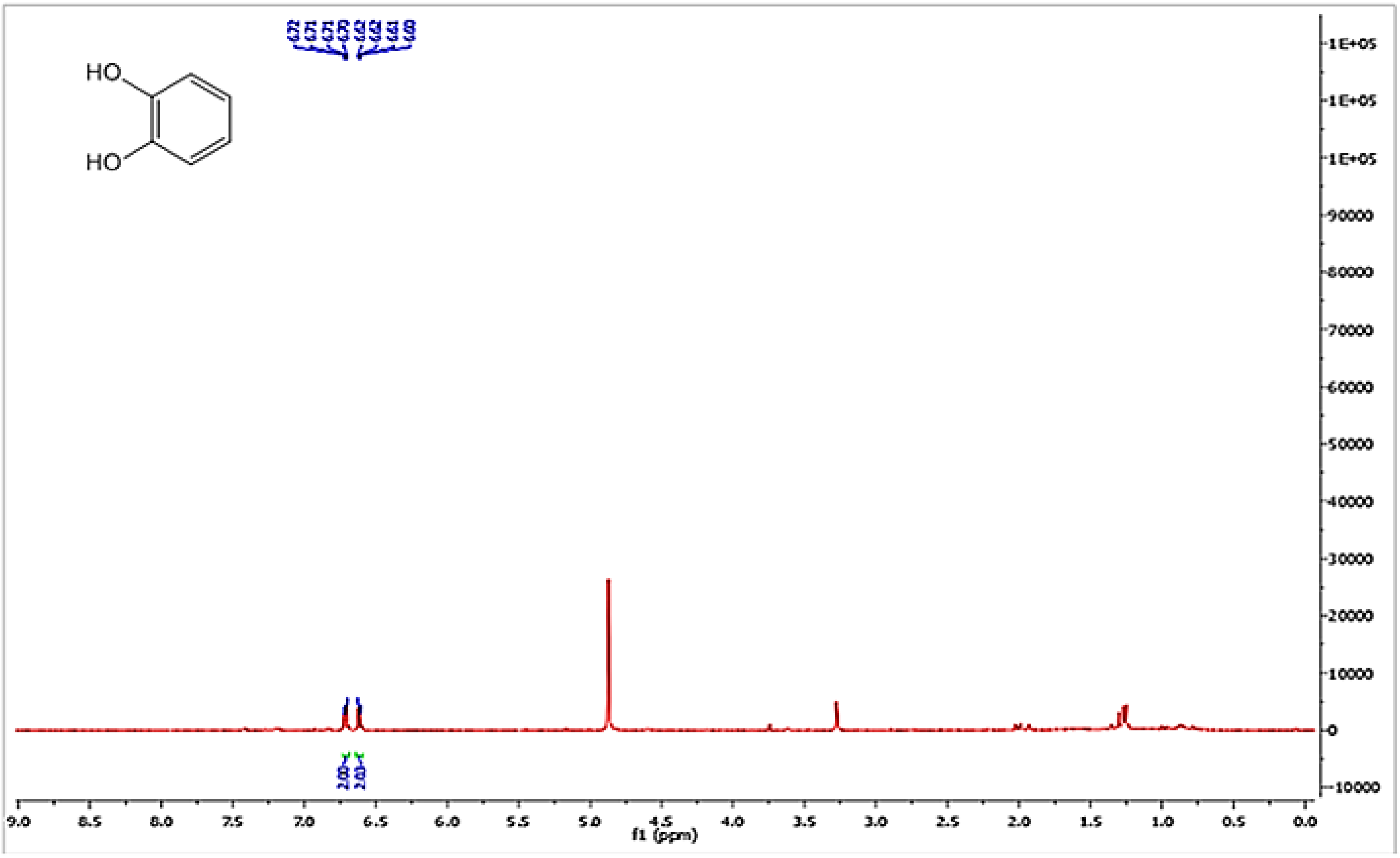

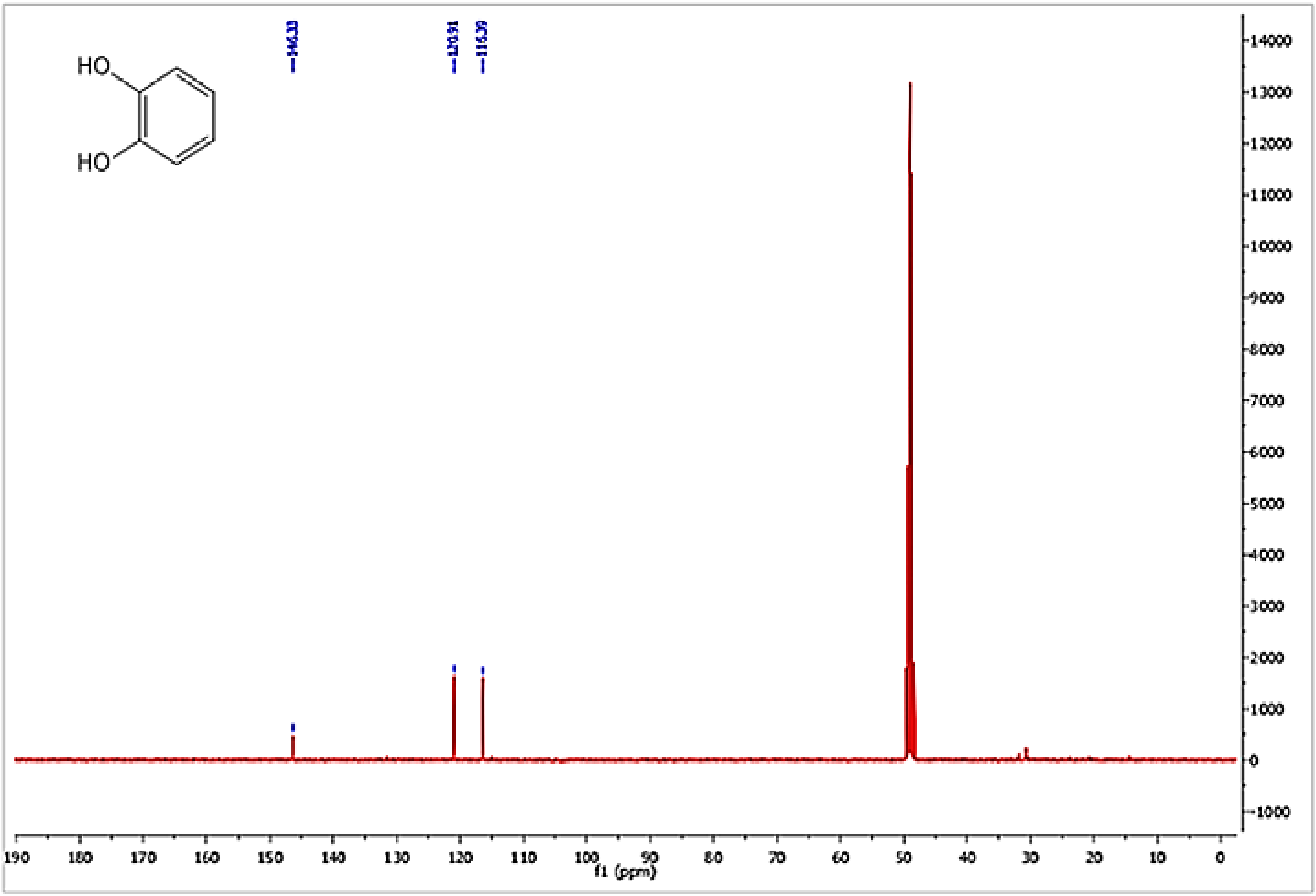
HO
HO
146.33
120.91
116.39
14000
13000
12000
11000
10000
9000
8000
7000
6000
5000
4000
3000
2000
1000
0
-1000
190 180 170 160 150 140 130 120 110 100 90 80 70 60 50 40 30 20 10 0
f1 (ppm)

^{13}C Attached Proton Test, CH_3/CH positive, CH_2/C negative (jmod)

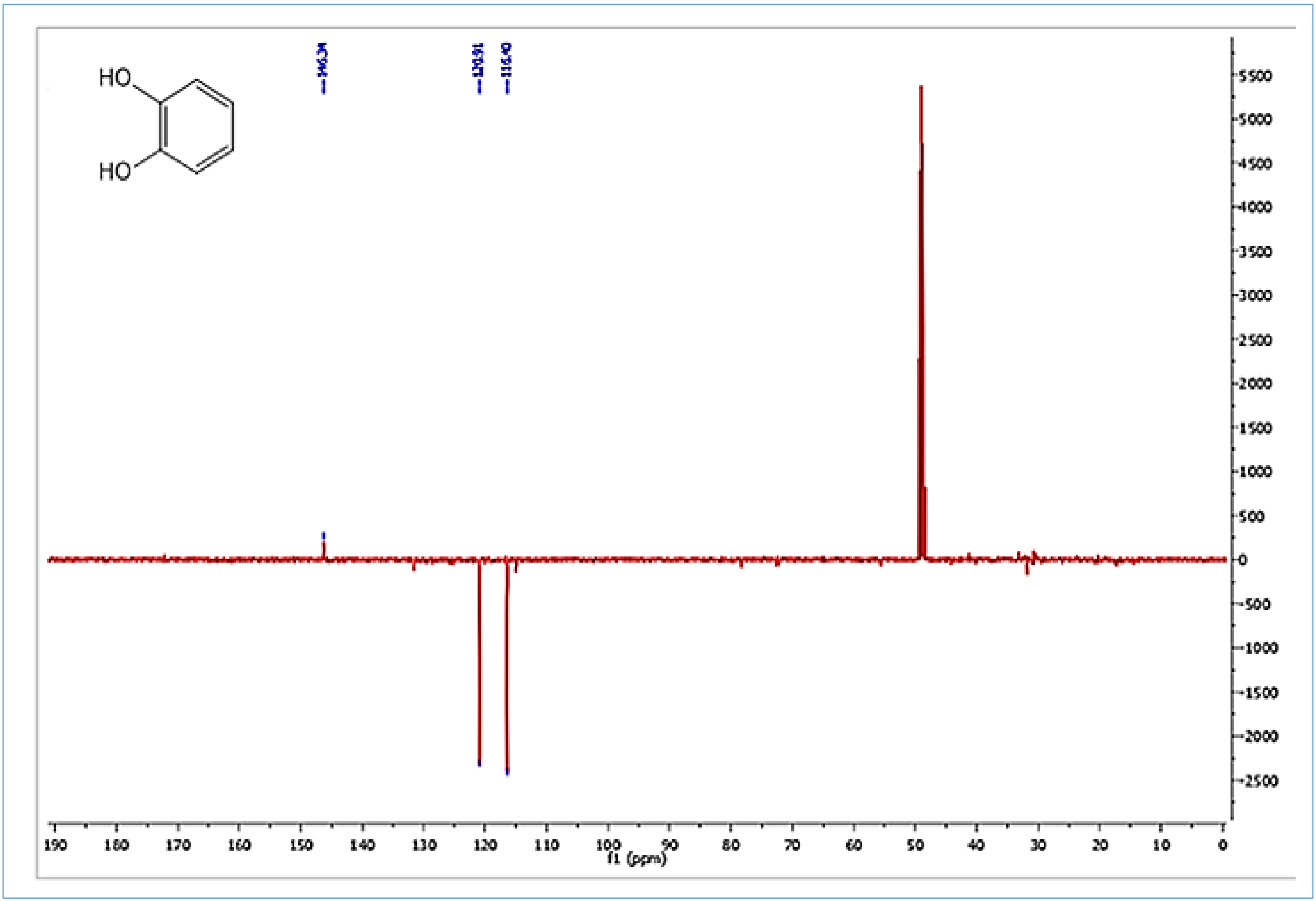

Protocatechuic acid (8)

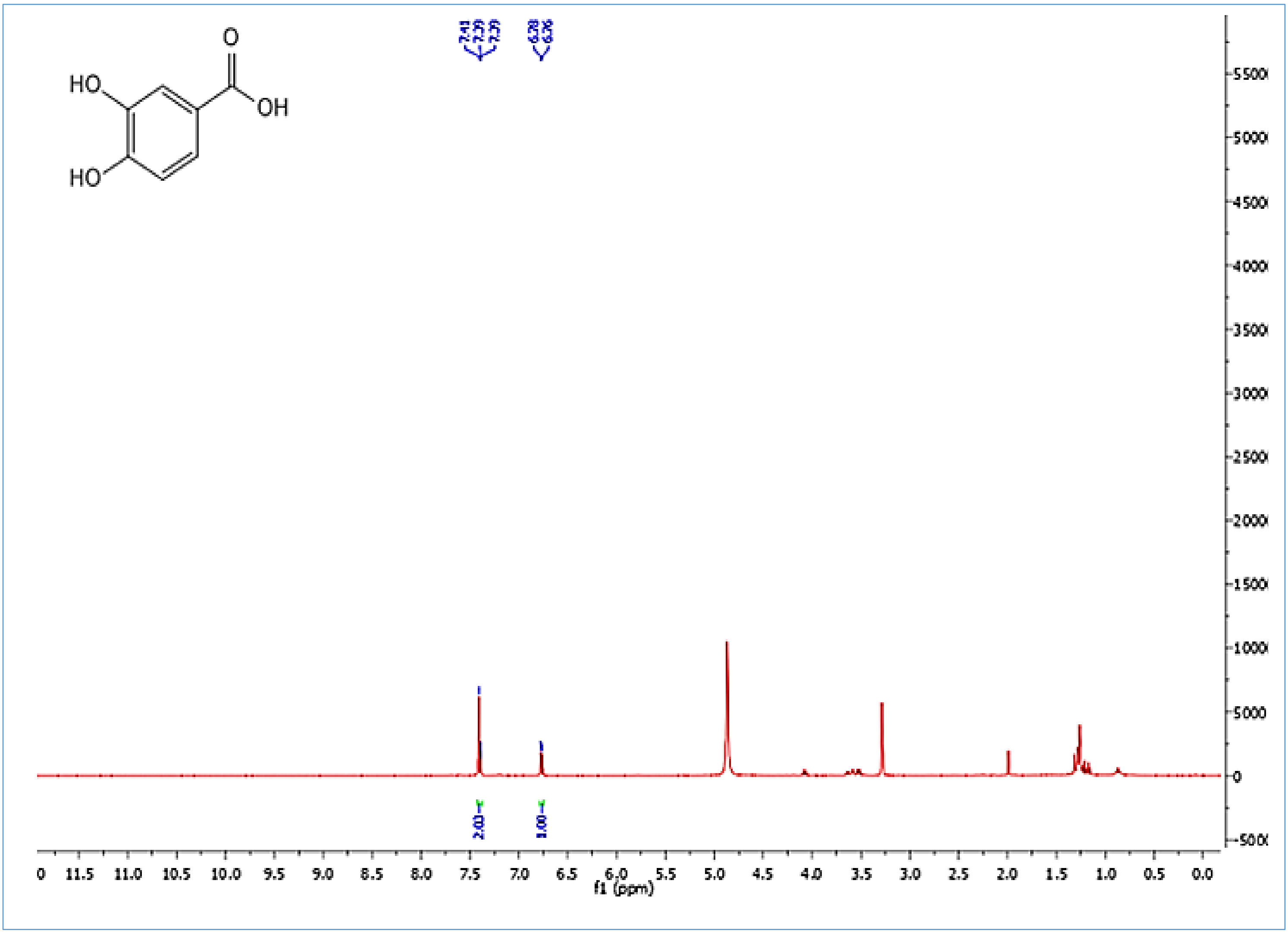

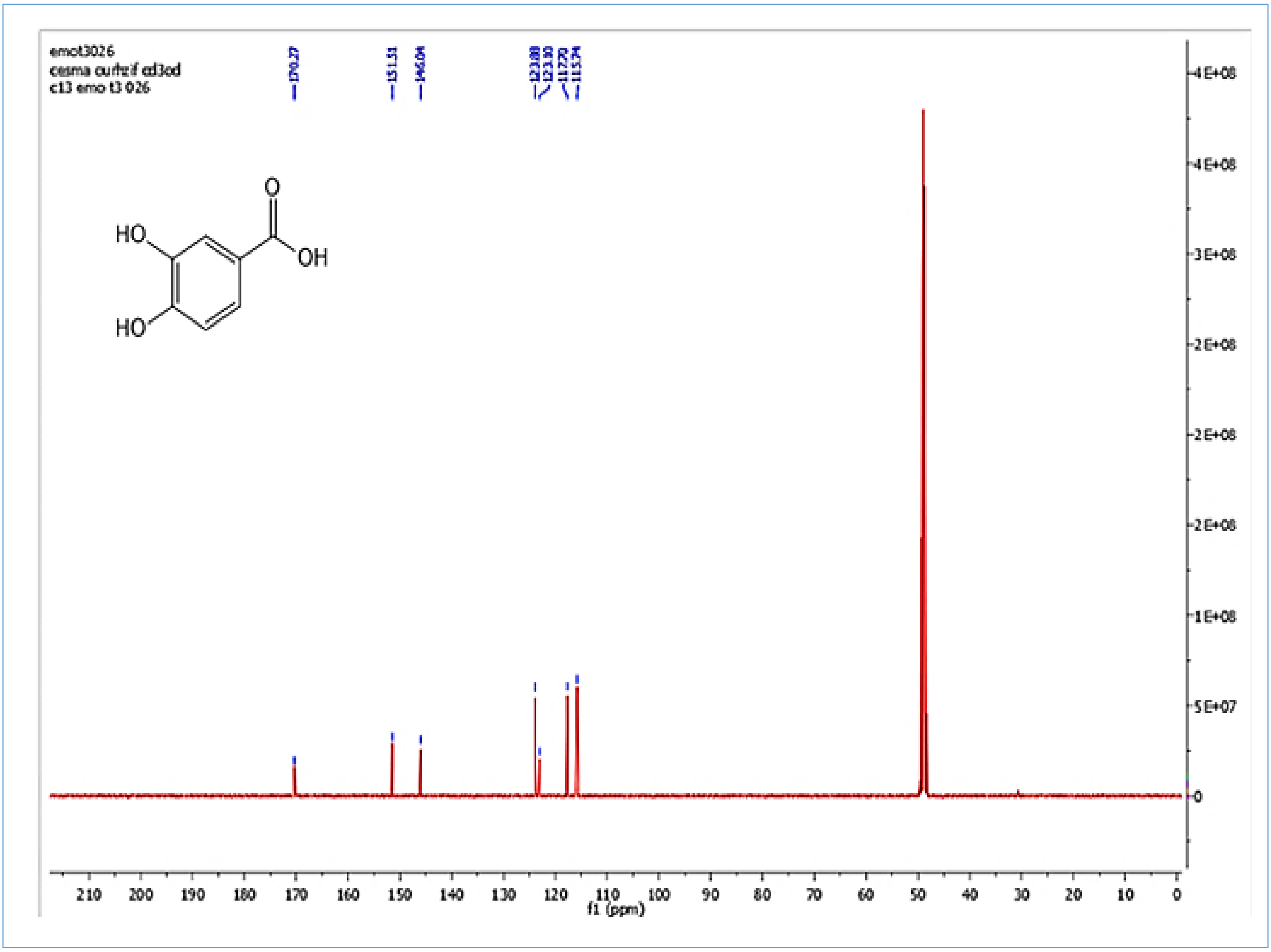
emot3026
cesma ourfz if cd3od
c13 emo t3 026
170.27
151.51
146.04
123.88
123.30
117.70
115.74
HO
HO
O
OH
f1 (ppm)

^{13}C Attached Proton Test, CH_3/CH positive, CH_2/C negative (jmod)

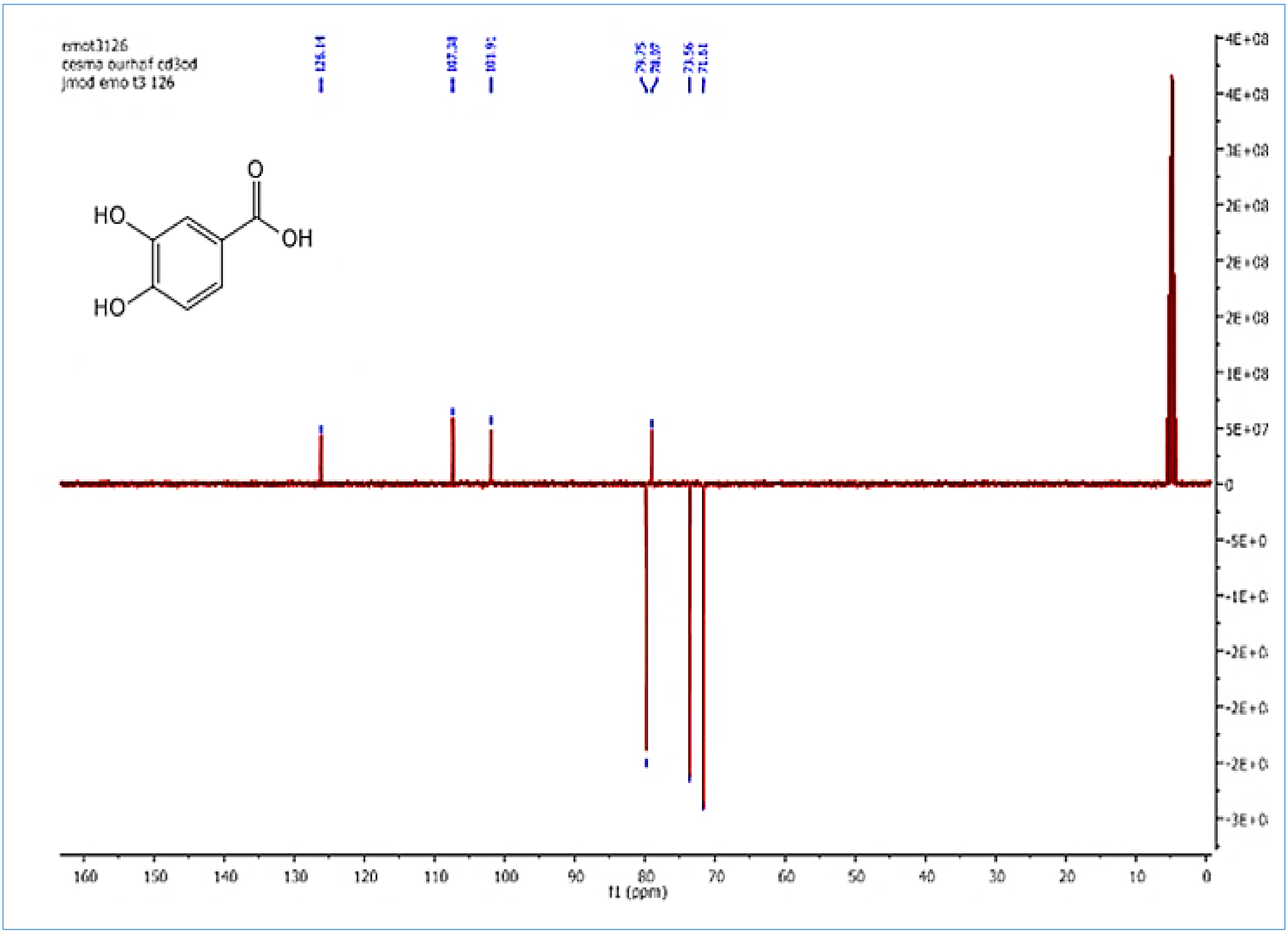

3,4-dihydroxyphenylacetic acid (9)

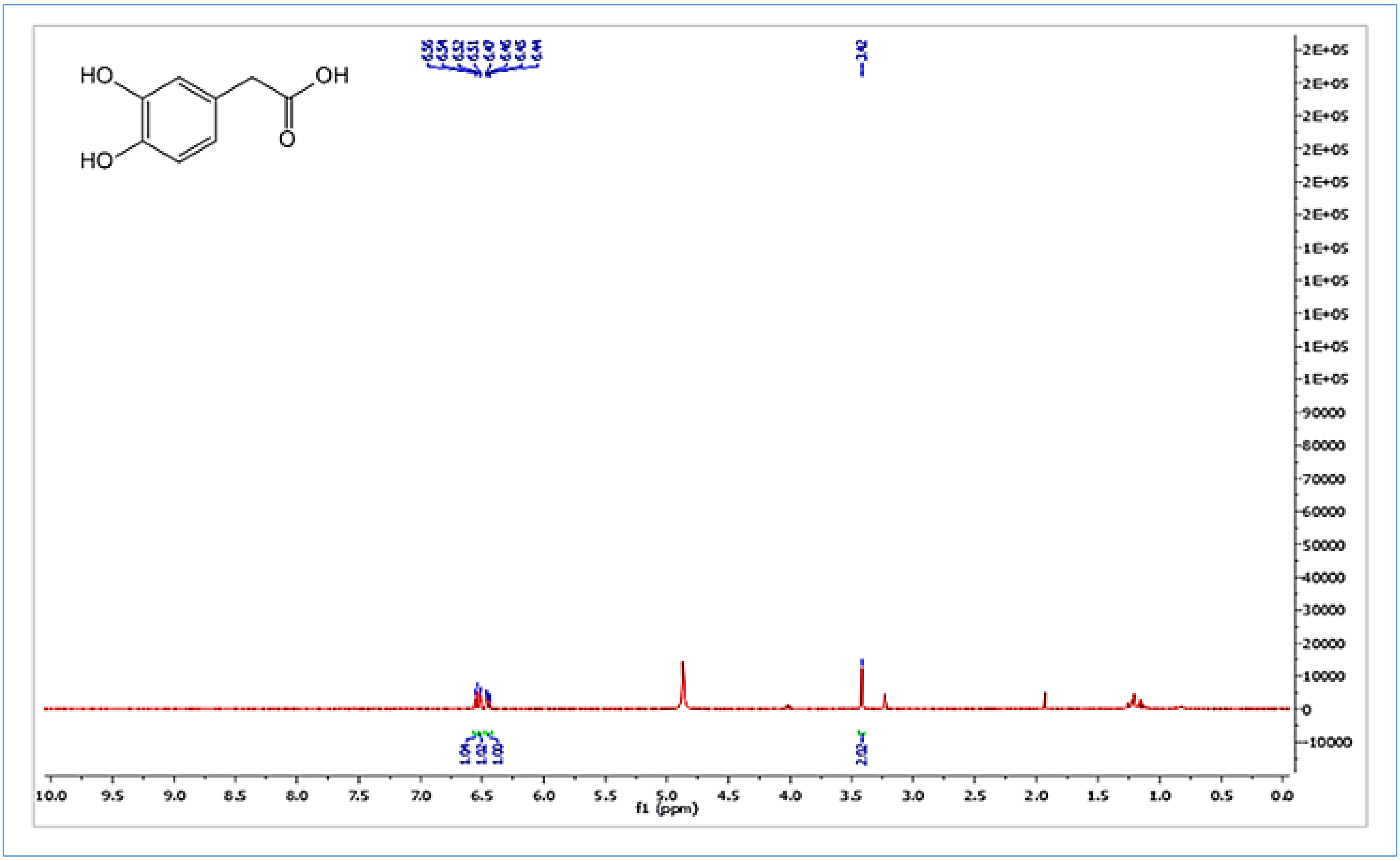

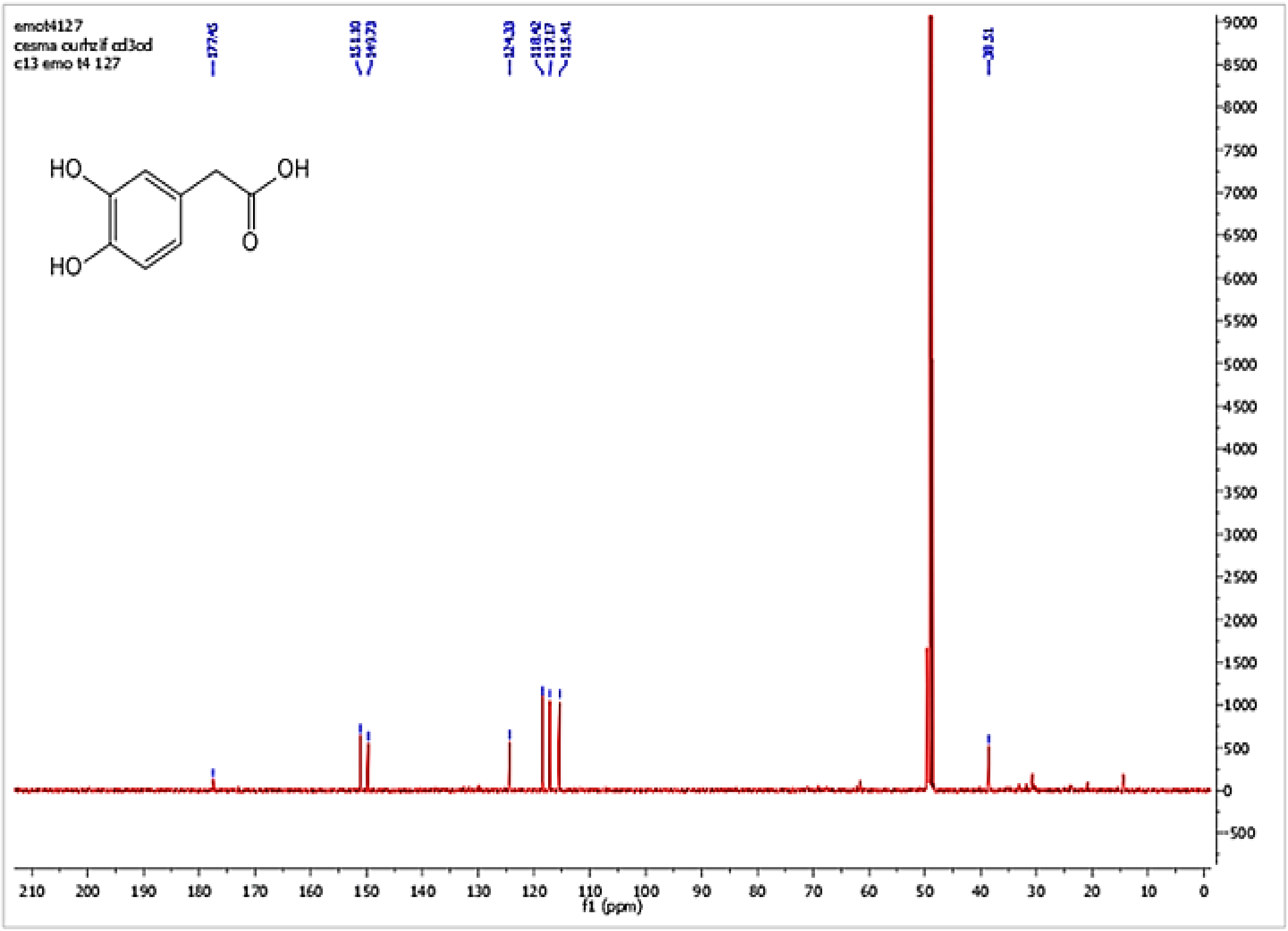
emot4127
cesma ourhz if cd3od
c13 emo t4 127
177.45
151.30
149.73
124.33
118.42
117.17
115.41
38.51
HO
HO
OH
O
f1 (ppm)
210 200 190 180 170 160 150 140 130 120 110 100 90 80 70 60 50 40 30 20 10 0
9000 8500 8000 7500 7000 6500 6000 5500 5000 4500 4000 3500 3000 2500 2000 1500 1000 500 0 -500

^{13}C Attached Proton Test, CH_3/CH positive, CH_2/C negative (jmod)

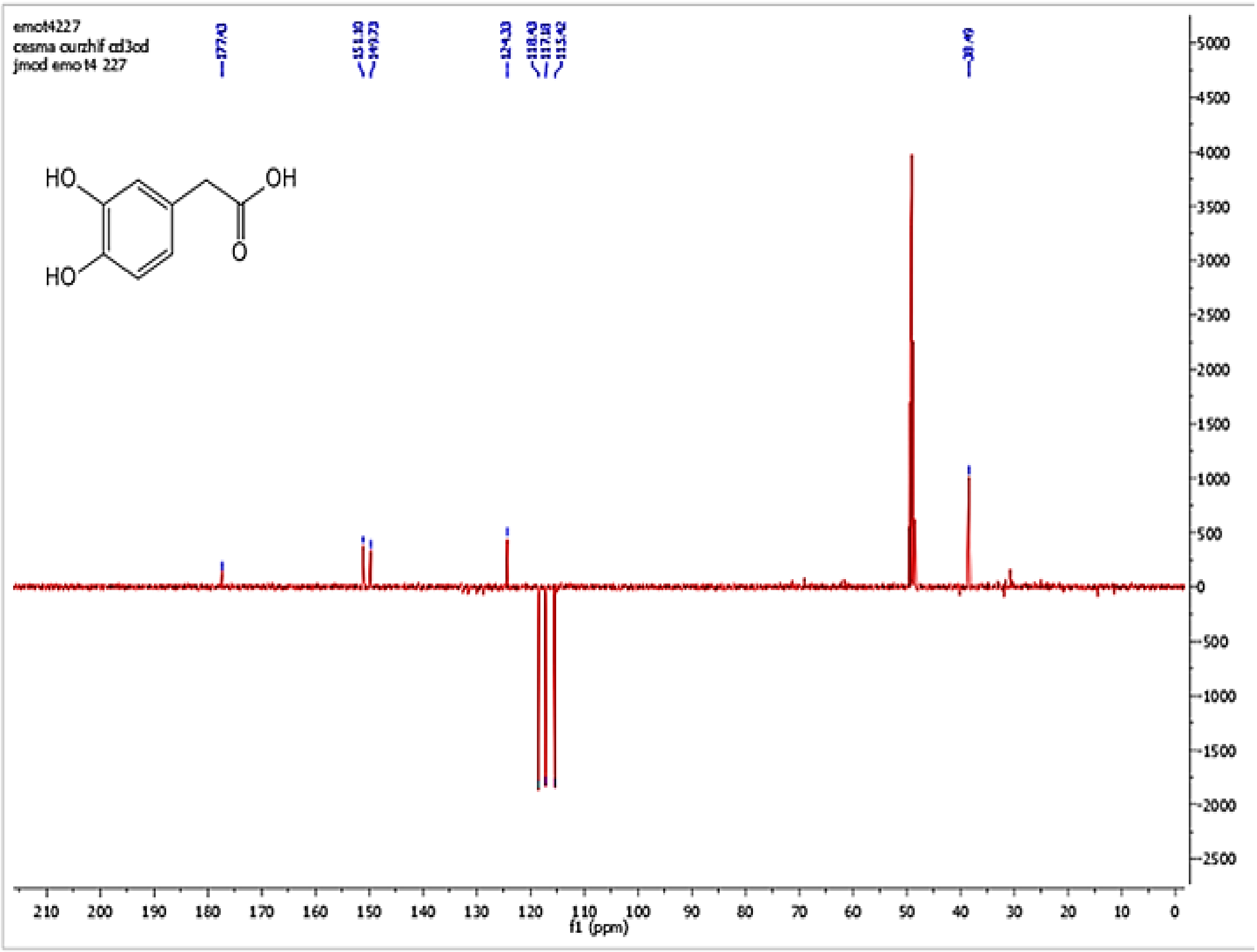

LC/HRMS SPECTRA:

12-Deoxyphorbol-13-angelate-20-acetate (1)

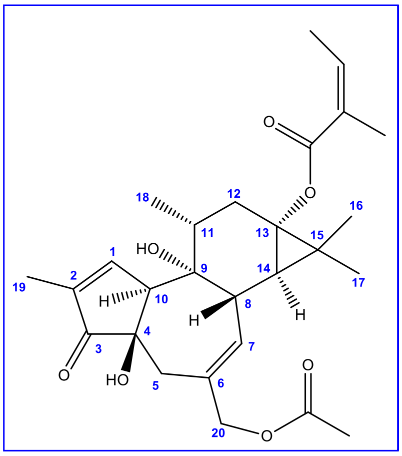

Chemical Formula: $C_{27}H_{36}O_7$

Molecular Weight: 472.57 g/mol

- **Negative ions**

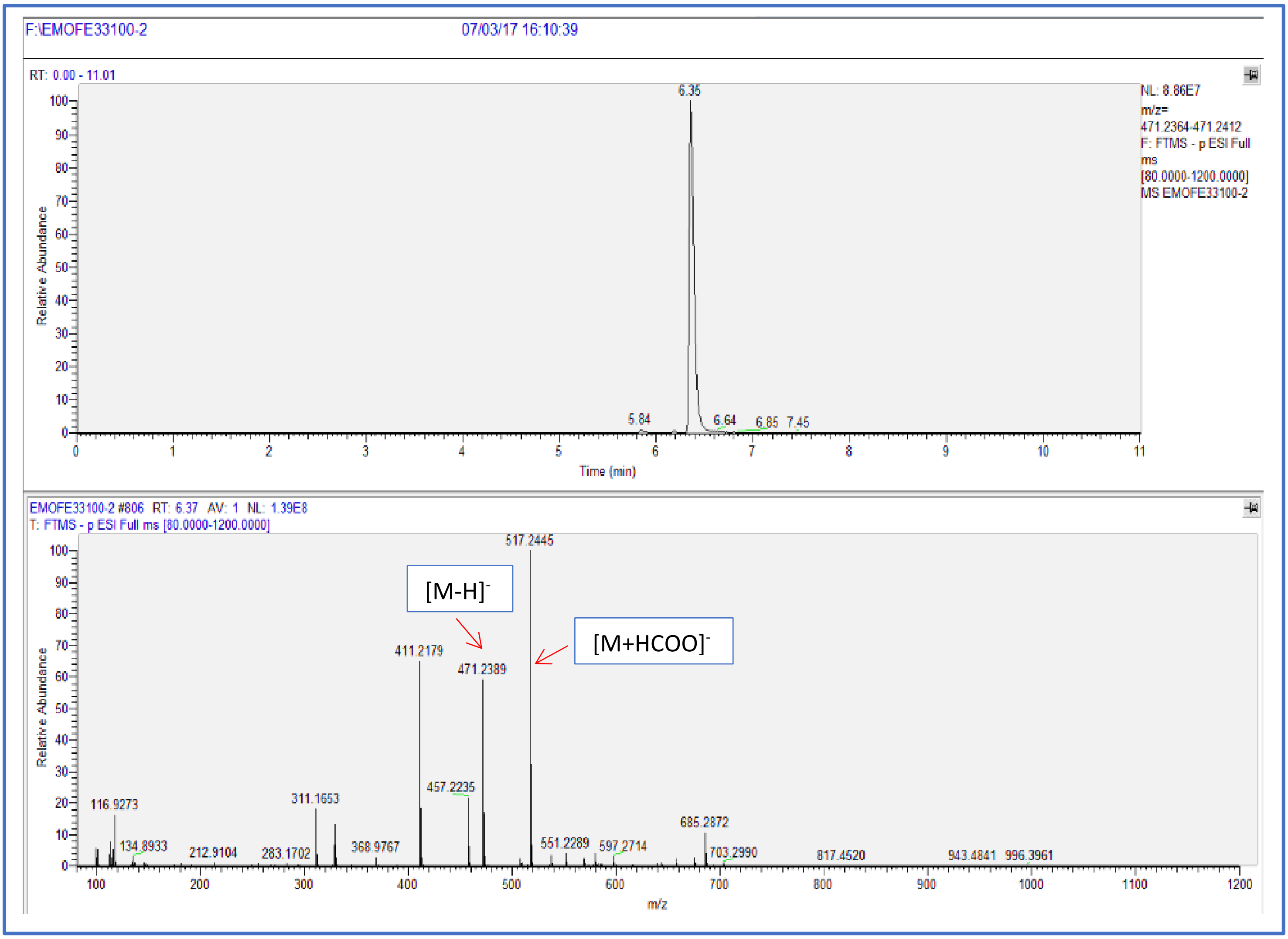

12-Deoxyphorbol-13-isobutyrate-20-acetate (2)

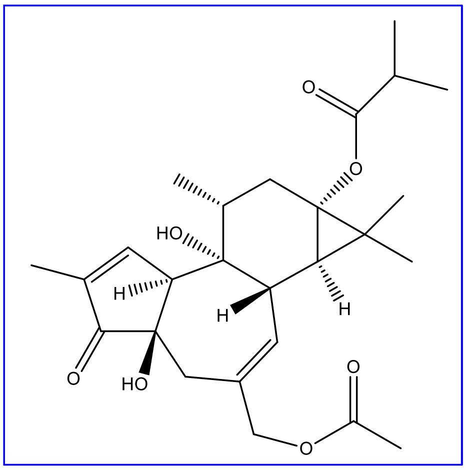

Chemical Formula: $C_{26}H_{36}O_7$

Molecular Weight: 460.56 g/mol

- **Negative ions**

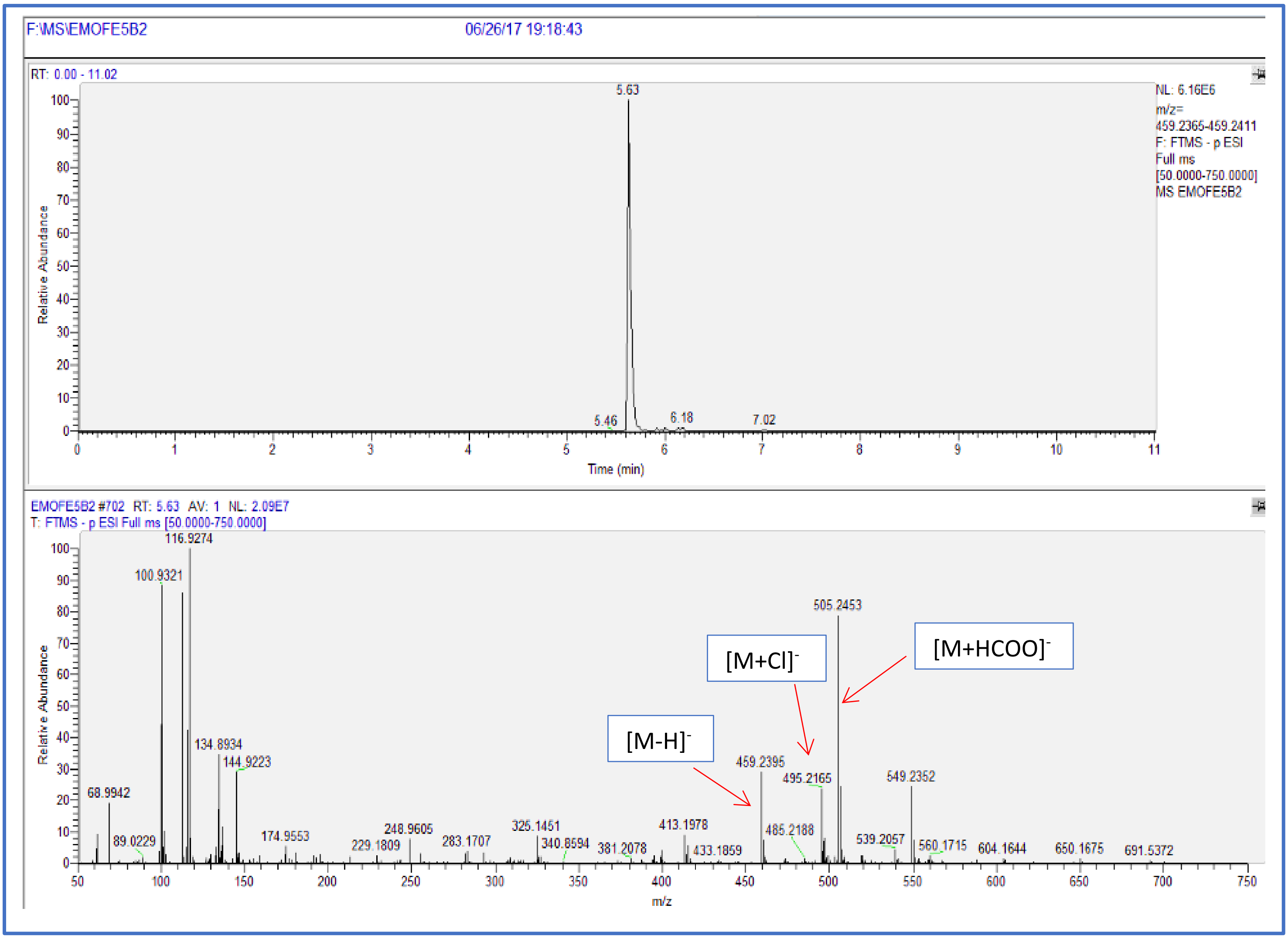

7-p-Métoxyphénylacétate-3,8,12-triacétate ingol (3)

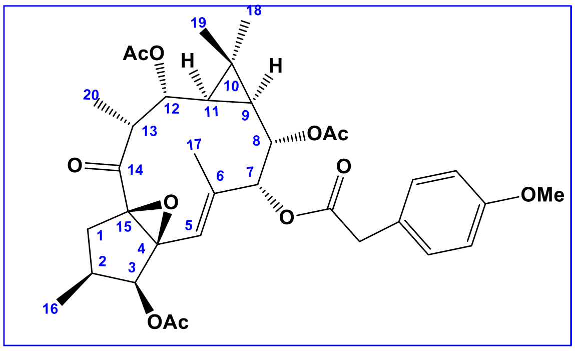

Chemical Formula: $C_{35}H_{44}O_{11}$

Molecular Weight: 640.72 g/mol

- **Negative ions**

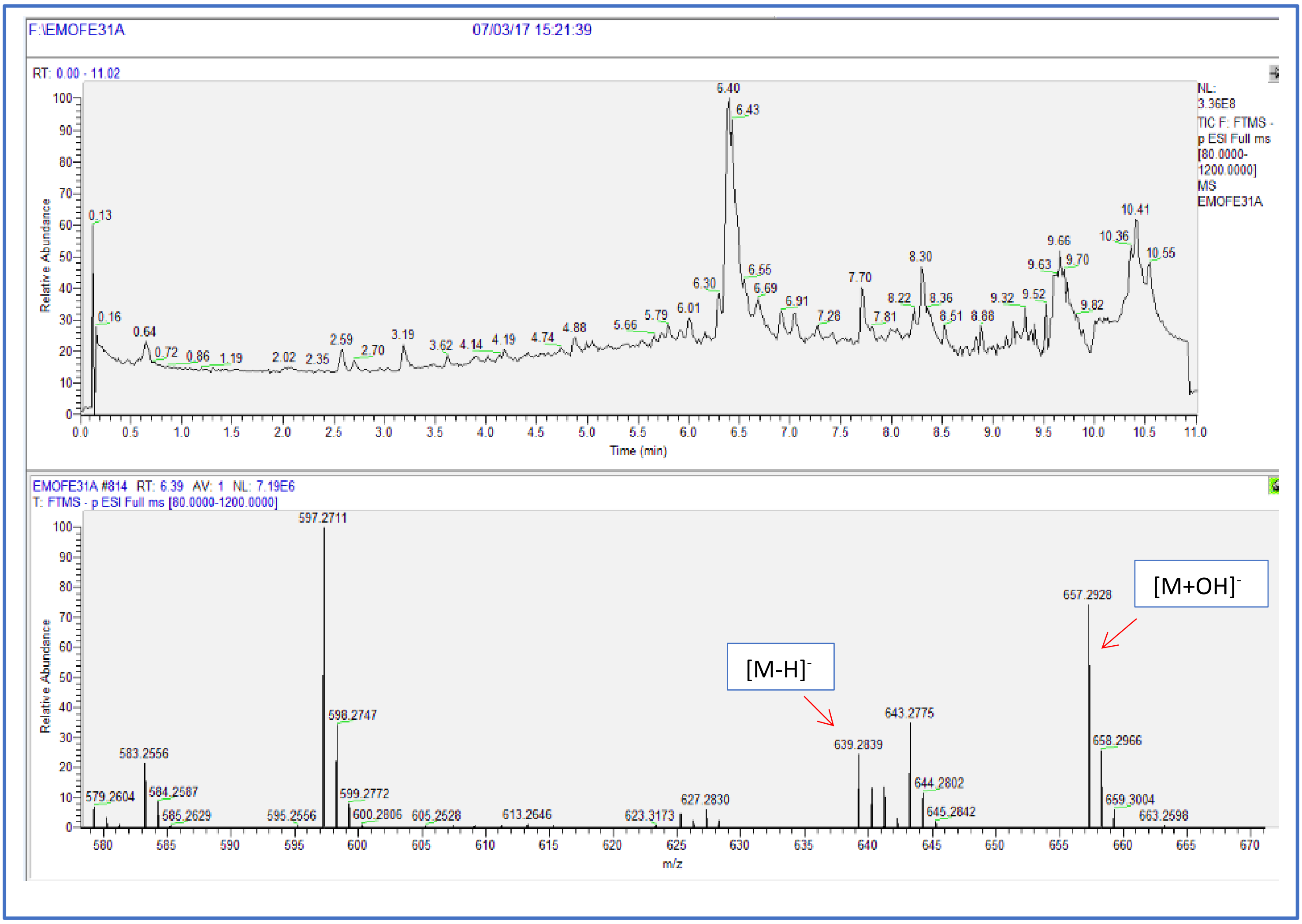

- **Positive ions**

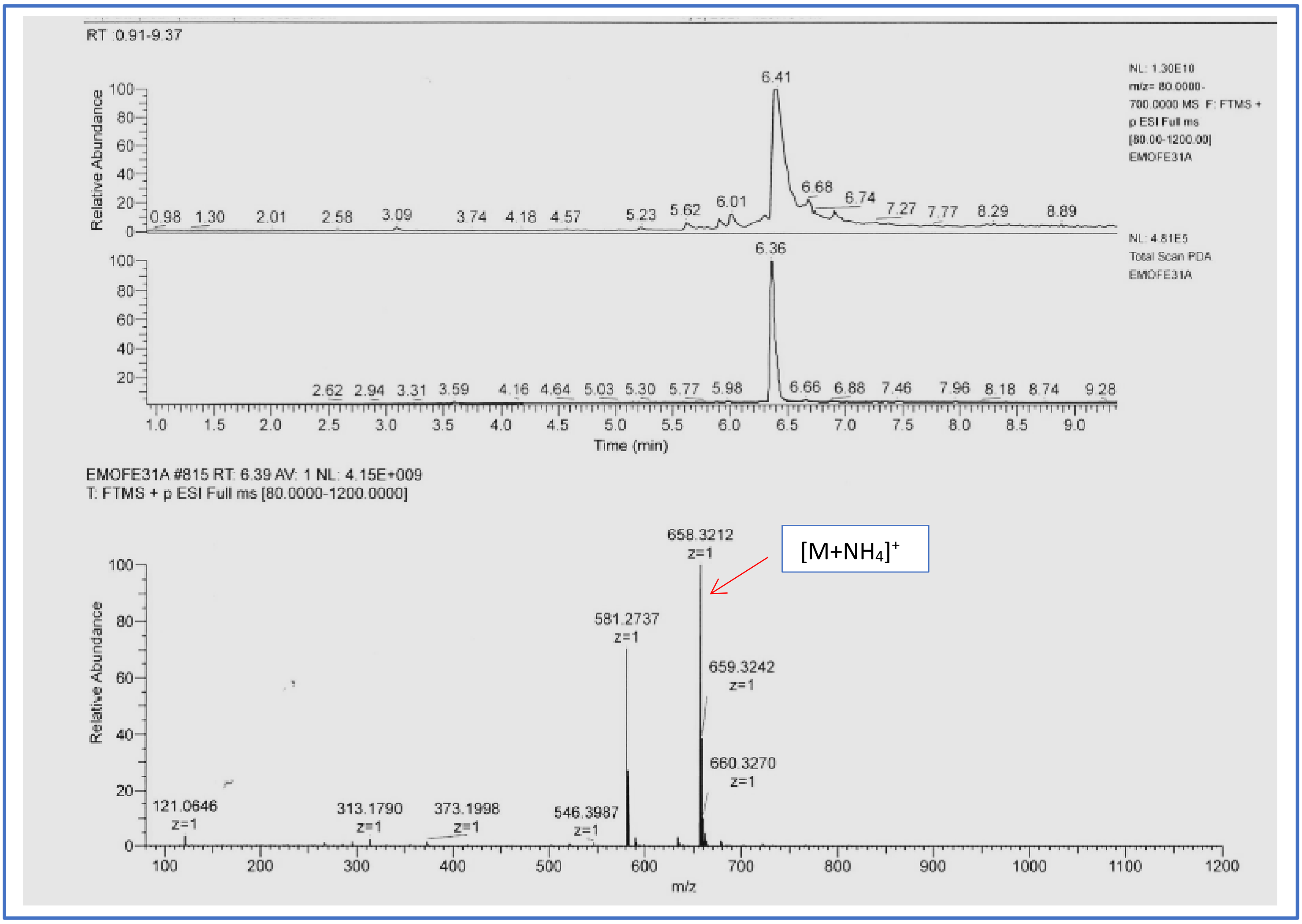

Resiniferatoxine (4)

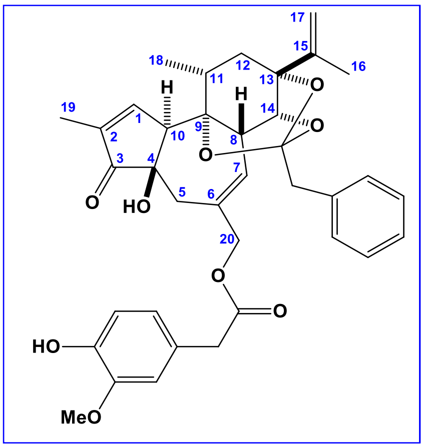

Chemical Formula: $C_{37}H_{40}O_9$

Molecular Weight: 628.27 g/mol

- **Positive ions**

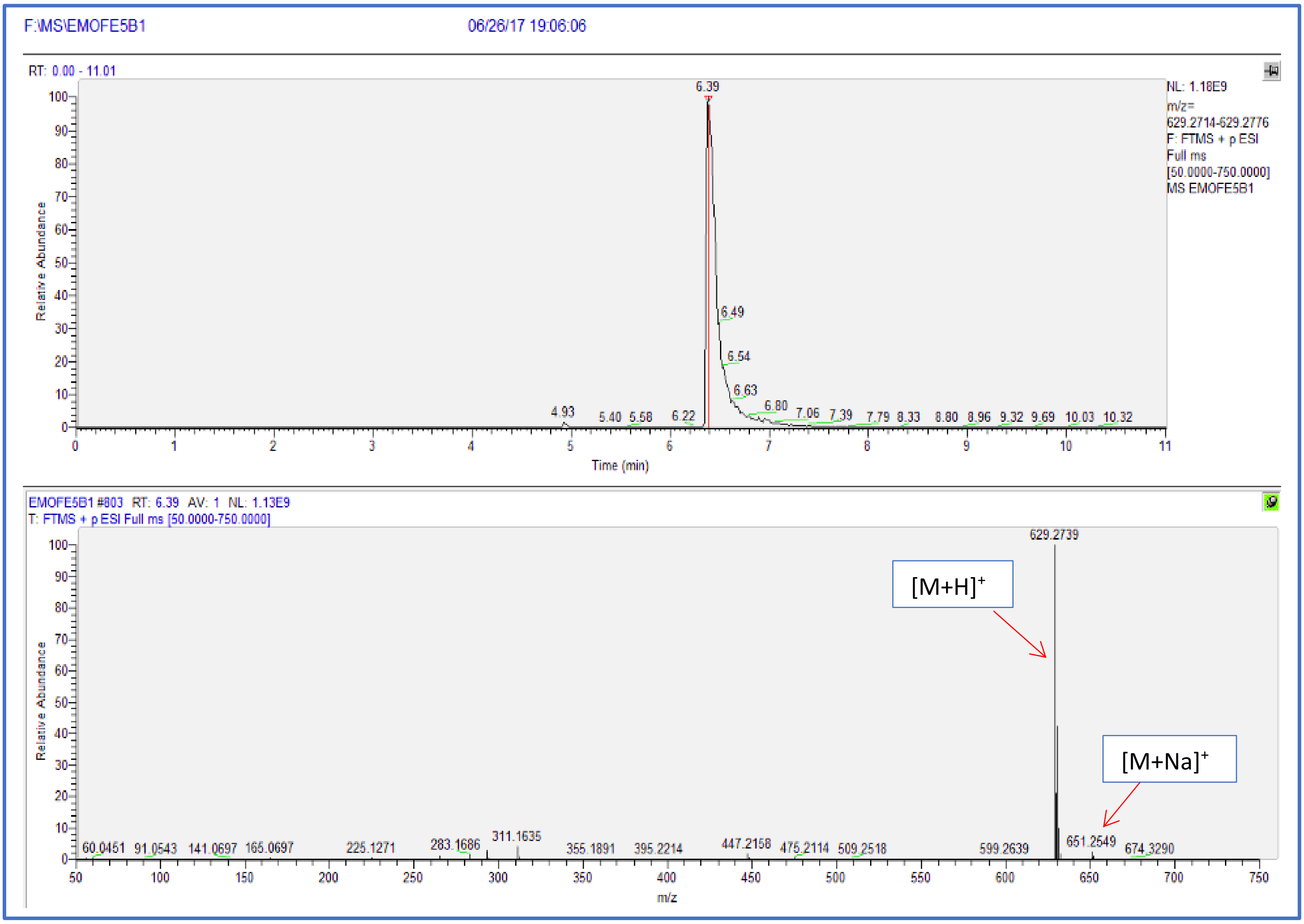

Deglucosyl euphorbioside A (5)

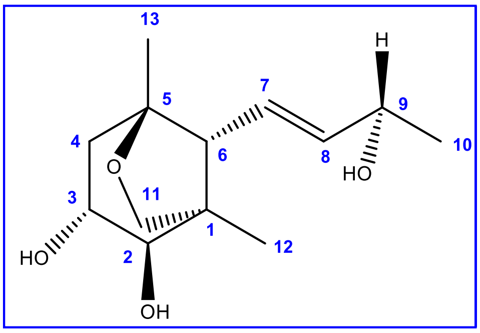

Chemical Formula: $C_{13}H_{22}O_4$

Molecular Weight: 242.31 g/mol

- **Positive ions**

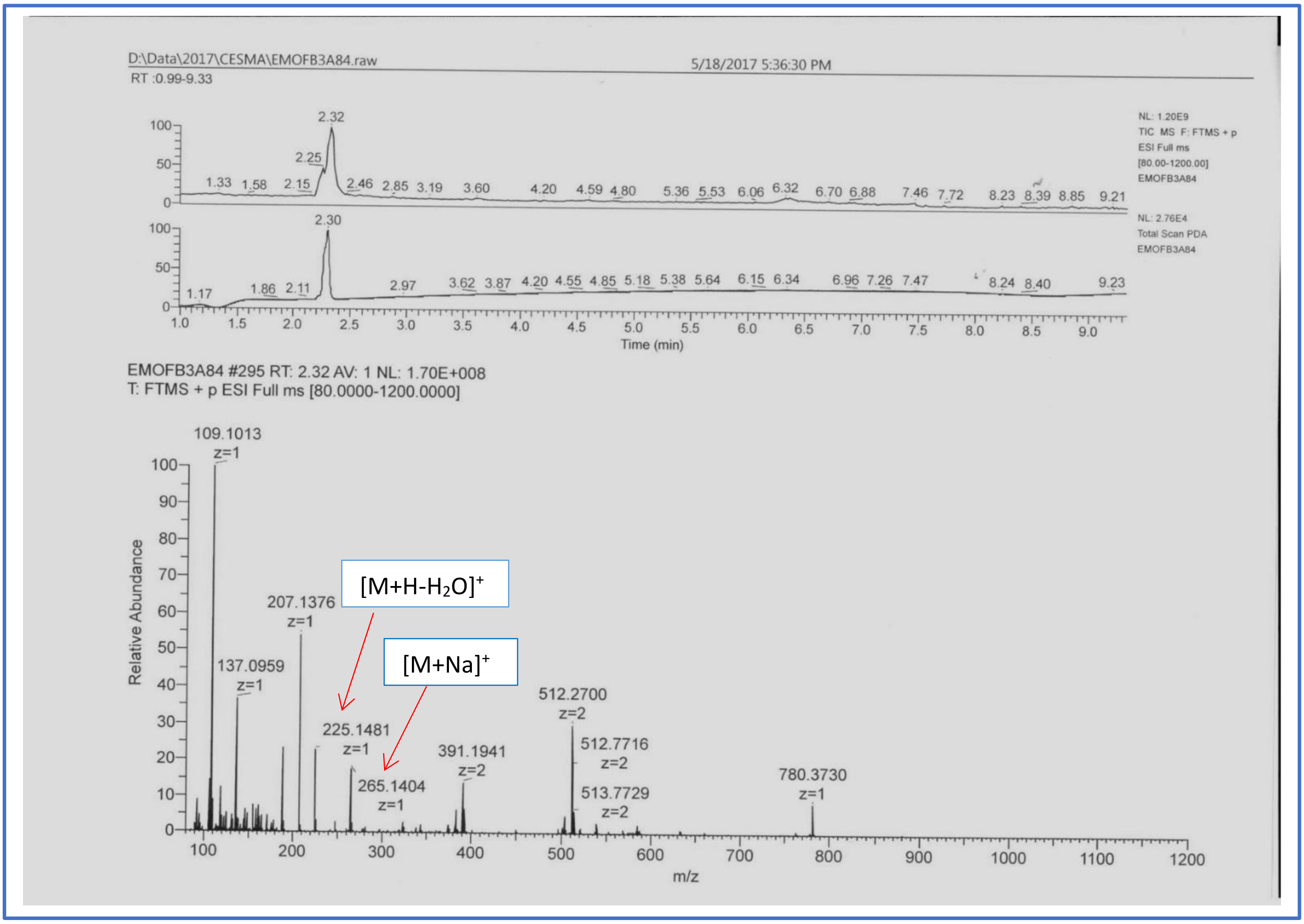

Euphorbioside A (6)

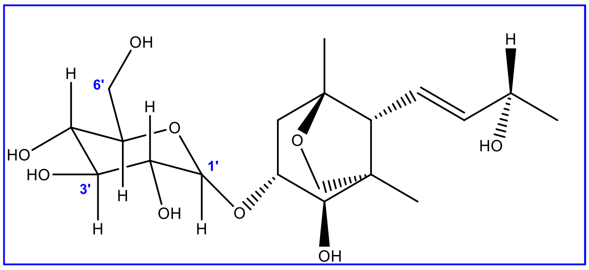

Chemical Formula: $C_{19}H_{32}O_9$

Molecular Weight: 404.45 g/mol

Positive ion

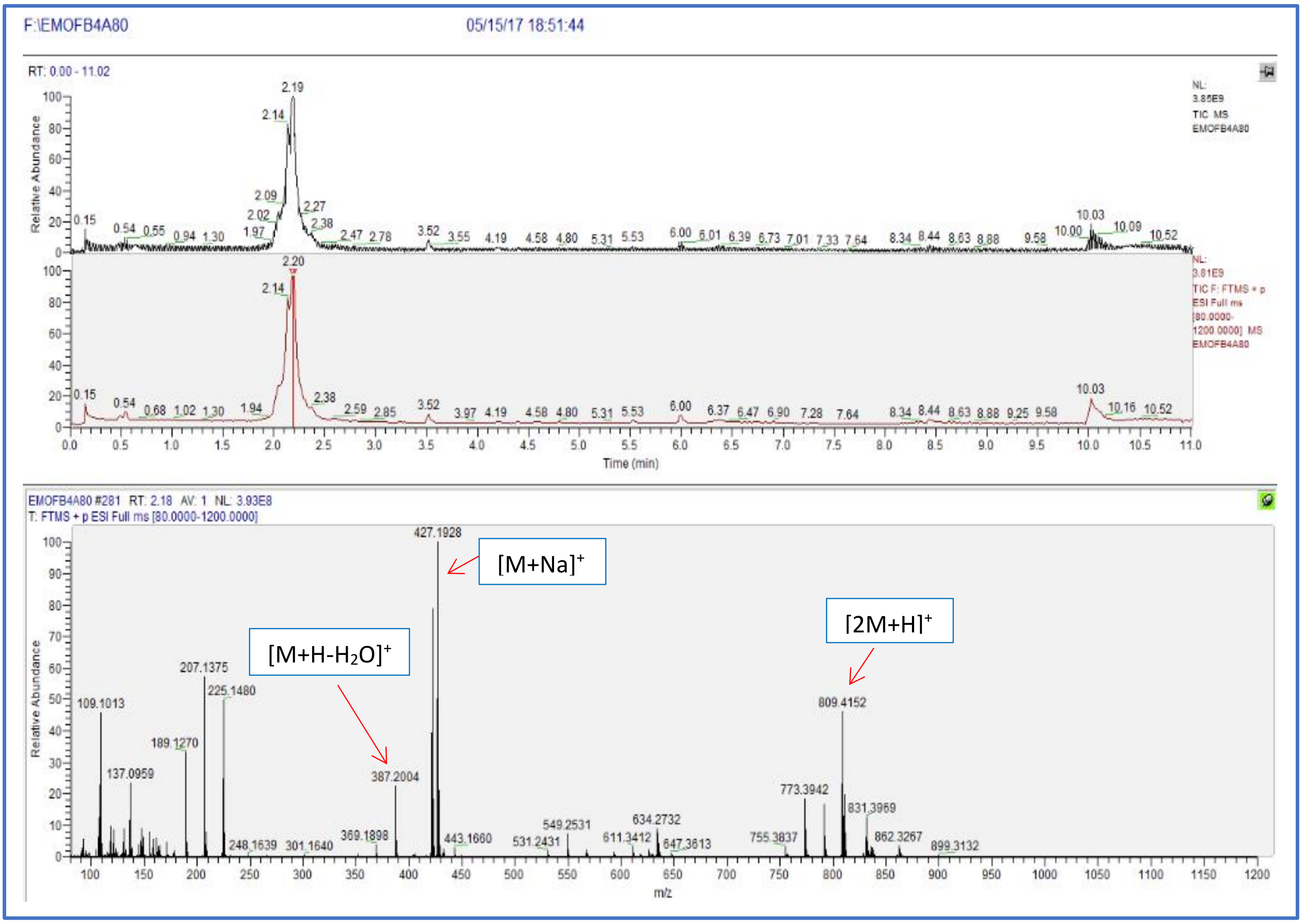

Catechol (7)

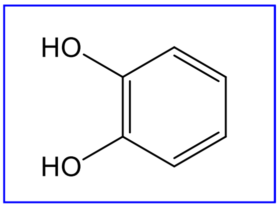

Chemical Formula: $C_6H_6O_2$

Molecular Weight: 110.11 g/mol

- **Negative ions**

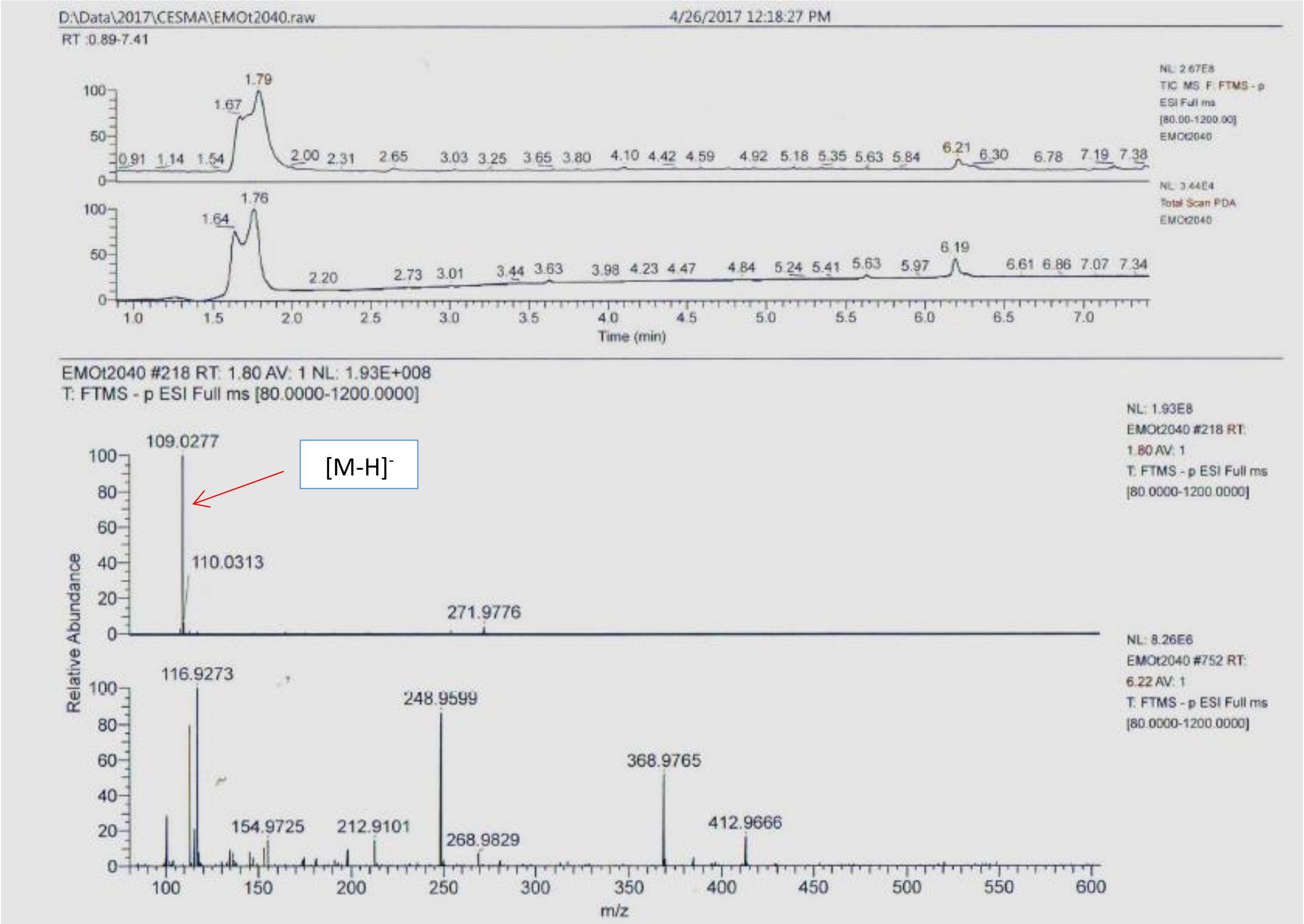

Protocatechuic acid (8)

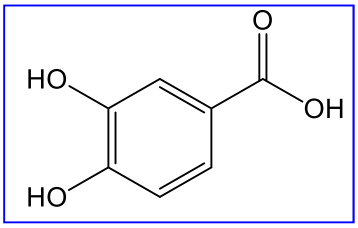

Chemical Formula: $C_7H_6O_4$

Molecular Weight: 154.12 g/mol

- **Negative ions**

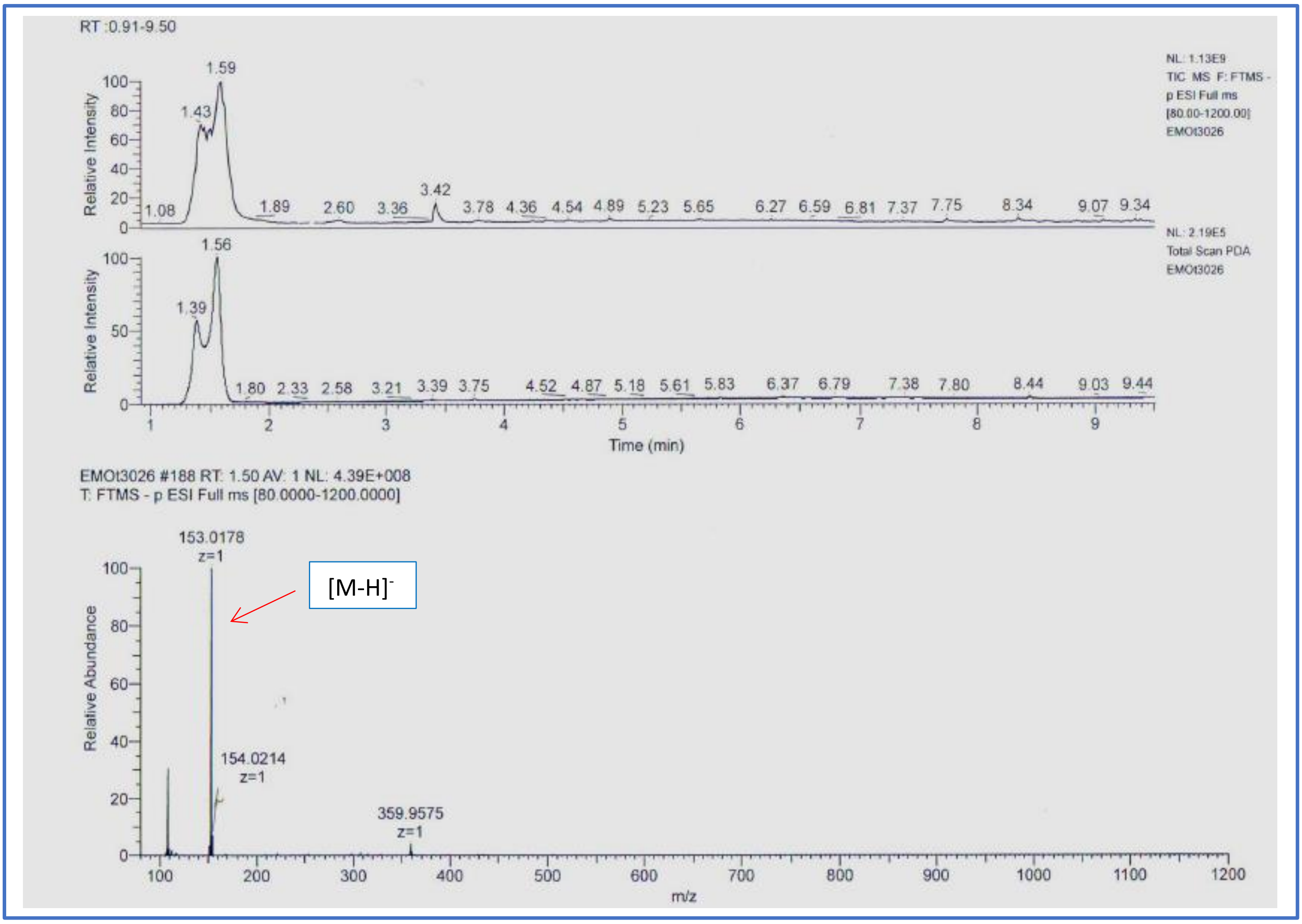

3,4-dihydroxyphenylacetic acid (9)

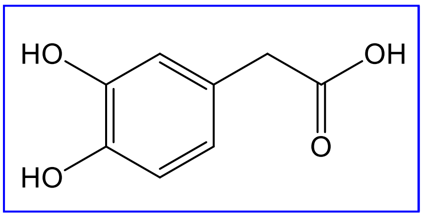

Chemical Formula: $C_8H_8O_4$

Molecular Weight: 168.15 g/mol

- **Negative ions**

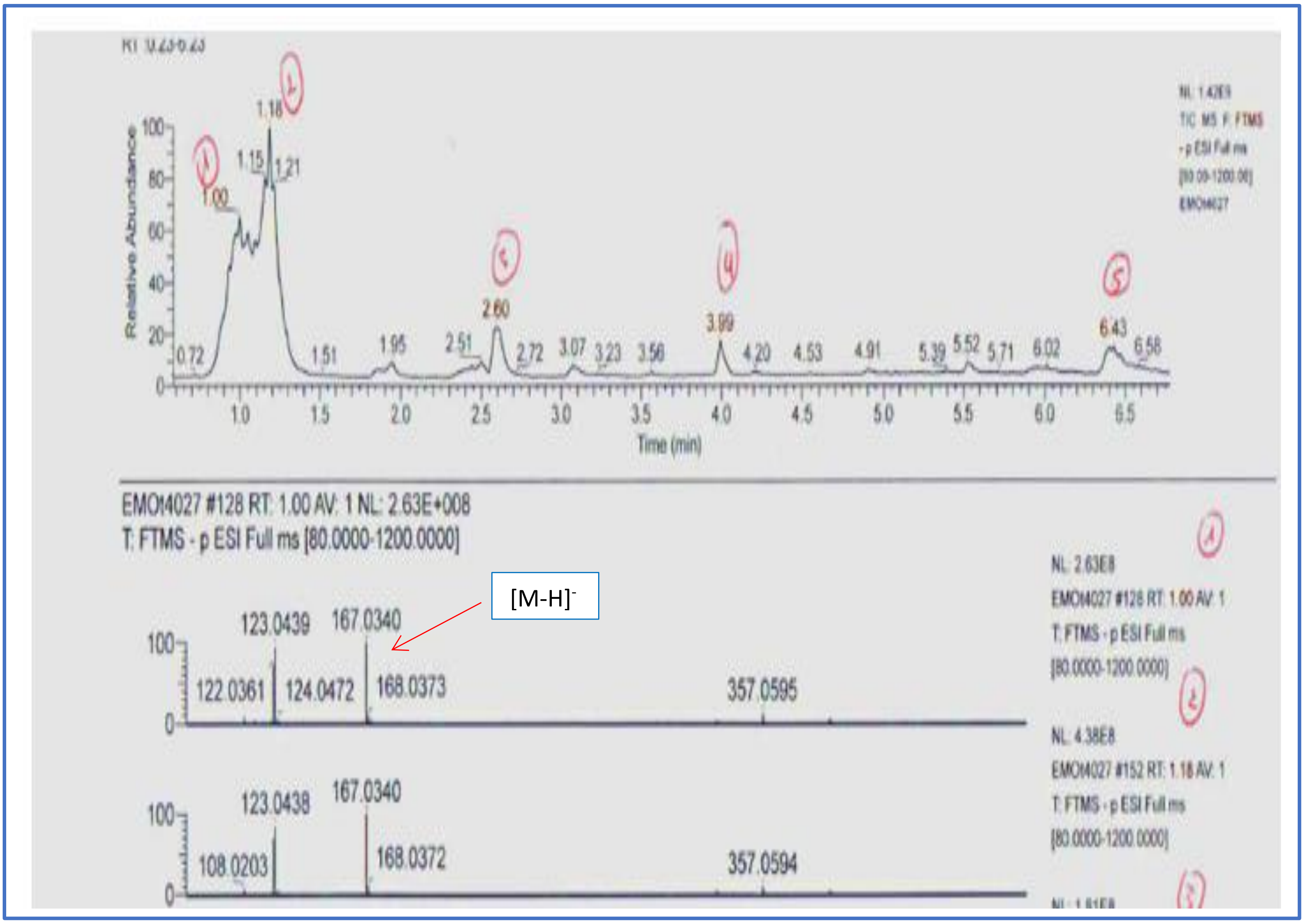

Supplement: Supplementary file 1 [file molecules-27-05234-s001.zip › molecules-1809648-supplementary.pdf]
